# Comparing efficacy of first-line treatment of metastatic castration resistant prostate cancer: a network meta-analysis of randomized controlled trials

**DOI:** 10.3389/fphar.2023.1290990

**Published:** 2023-11-22

**Authors:** Yang Liu, Xianzhong Deng, Zhi Wen, Jing Huang, Chongjian Wang, Caixia Chen, Erhao Bao, Jiahao Wang, Xuesong Yang

**Affiliations:** ^1^ Department of Urology, Affiliated Hospital of North Sichuan Medical College, Nanchong, China; ^2^ Department of Urology, Chengdu Xinhua Hospital Affiliated to North Sichuan Medical College, Chengdu, China

**Keywords:** prostate cancer, castration-resistant prostate cancer, metastatic, PARP, targeted therapy, meta-analysis

## Abstract

**Background:** Metastatic castration-resistant prostate cancer (mCRPC) presents significant treatment selection challenges due to limited therapeutic options. This study aimed to comprehensively assess the efficacy of multiple treatment regimens for mCRPC through a network meta-analysis (NMA) of randomized controlled trials (RCTs).

**Methods:** A systematically comprehensive search for randomized controlled trials (RCTs) was performed in Pubmed, Cochrane Library, Embase, and Web of Science databases. The network meta-analysis was employed to compare the overall survival (OS), progression-free survival (PFS), and radiographic progression-free survival (rPFS) among different interventions at specific time points. This study was prospectively registered with PROSPERO (CRD42023422823).

**Results:** A total of 29 RCTs, involving 12,706 patients and investigating 16 interventions, were included in the analysis. Chempretarget ((capivasertib or cabozantinib) + docetaxel + prednisone)) and PARP (Olaparib or rucaparib) inhibitors emerged as interventions that significantly improved survival outcomes compared to first-line treatment in mCRPC patients. Chempretarget demonstrated superior overall survival starting from the 12th month, while PARP inhibitors showed a clear advantage in progression-free survival within the 3–18 months range. Notably, chempre ((Docetaxel or Cabazitaxel) + prednisone) exhibited favorable performance in radiographic progression-free survival during the 3–18 month period.

**Conclusion:** Our findings underscore the efficacy of chempretarget, PARP inhibitors, and chempre in enhancing survival outcomes for mCRPC patients. Further head-to-head comparisons are warranted to validate these results. These findings carry important implications for treatment decision-making in mCRPC and may guide the development of more effective therapeutic strategies.

## Introduction

Prostate cancer ranks among the most prevalent malignancies in men, second only to lung cancer. Over the period from 2014 to 2019, the United States is projected to witness an annual increase of 3% in the incidence rate of prostate cancer, leading to the emergence of 99,000 new cases annually (Siegel et al., 2023). Castration-resistant prostate cancer (CRPC) refers to the radiological or biochemical progression of prostate cancer despite standard androgen deprivation therapy (ADT) when serum testosterone levels have reached castration levels (testosterone levels less than 50 ng/dl or 1.7 nmol/L) (Zarour and Alumkal, 2010/5). Median survival for CRPC stands at approximately 14 months, with a range of 9–30 months (Kirby et al., 2011/11). Furthermore, around 2 years after the onset of CRPC, 15%–33% of patients will experience metastasis, leading to a significant escalation in mortality rates (Hirst et al., 2012/12; Smith et al., 2005/5).

Given the aggressive nature of metastatic castration-resistant prostate cancer, the treatment options available for this disease remain limited. Recently, numerous well-designed RCTs have investigated multiple treatment approaches with the aim of enhancing outcomes for patients with mCRPC. These approaches encompass castration therapy, Poly ADP Ribose Polymerase (PARP) inhibitors, programmed cell death protein 1 (PD-1) inhibitors, and chemotherapy. The findings have demonstrated that castration therapy, including PARP inhibitors have shown encouraging anticancer activity (Teyssonneau et al., 2021; Bieńkowski et al., 2022). PD-1 inhibitors and chemotherapy have also exhibited positive effects on survival (Nuhn et al., 2019; Merseburger et al., 2021).

Given the diverse range of treatment options available for metastatic castration-resistant prostate cancer, it is crucial to assess their relative efficacy and identify the optimal treatment strategy. Although previous meta-analyses have examined comparisons of specific treatment types, such as between targeted drugs or between PARP inhibitors (Poorthuis et al., 2017; Wang et al., 2018; Rizzo et al., 2022), as far as we are aware, no study has comprehensively evaluated combined or isolated comparisons across multiple treatment modalities.

Therefore, this study aimed to conduct a network meta-analysis of RCTs to evaluate the efficacy of different treatment regimens for metastatic castration-resistant prostate cancer. The results of this analysis will enhance our understanding of the relative effectiveness of these treatments and provide valuable information to guide clinicians and patients in making informed decisions about the most appropriate treatment.

## Materials and methods

The present meta-analysis adhered to the guidelines outlined in the Preferred Reporting Items for Systematic Reviews and Meta-Analyses (PRISMA) statement (Shamseer et al., 2015) and was prospectively registered with PROSPERO (ID: CRD 42023422823).

### Literature search strategy

We conducted a comprehensive search in multiple databases including Cochrane Library (CENTRAL), PubMed, Web of science and Embase. The search spanned from the inception of these databases to 30 May 2023, and utilized specific MeSH terms as follows: “castration resistant prostate cancer”, “mcrpc”, “castration-resistant prostate cancer”, “carcinoma”, “tumor”, “Docetaxel”, “Cabazitaxel”, “Mitoxantrone”, “Platinum-based chemotherapy”, “Abirateone”, “Enzalutamide”, “Apalutamide”, “PARP”, “Olaparib”, “Niraparib”, “Rucaparib”, “Veliparib”, “Talazoparib”, “PD-1”, “pembrolizumab”, “CTLA4”, “ipilimumab”, “Ipatasertib”, and “random”. Furthermore, we performed a manual search and review of relevant references to ensure comprehensive coverage and minimize the risk of omitting any relevant studies. Only studies published in English were included in the reference list.

### Study selection

The PICOS approach was used to define the inclusion criteria (Siegel et al., 2023). The mCRPC patient population (Zarour and Alumkal, 2010/5); experience any systemic treatment within 6 months (Kirby et al., 2011/11); Patients were treated with the following drugs alone or in combination: Abiraterone, Enzalutamide, olaparib, Docetaxel, Cabazitaxel, DCVAC, ipatasertib, carboplatin, capivasertib, cabozantinib, ipilimumab, atezolizumab, tivantinib, rucaparib, buparlisib, Orteronel (Hirst et al., 2012/12); one or more of the following outcomes:OS, PFS or rPFS; and (Smith et al., 2005/5) RCTs.

Following are the exclusion criteria (Siegel et al., 2023):observational studies (Zarour and Alumkal, 2010/5); conference abstract, review or letters observational studies (Kirby et al., 2011/11);studies with unavailable data for analysis (Hirst et al., 2012/12);a comparative study between a class of drugs and (Smith et al., 2005/5) non-English literature.

### Data extraction and quality assessment

For the included studies, two investigators (YL and DX) independently extracted the data. The Cochrane Risk of Bias 2.0 tool was utilized to assess the risk of bias for each randomized controlled trial (RCT), and any discrepancies were resolved through arbitration by a senior reviewer (YX). The following variables were recorded: first author’s name, country of study, publication year, number of patients, drug type, therapeutic drugs, median follow-up time, hazard ratios (HR) and 95% confidence intervals (CI) associated with progression-free survival (PFS), radiographic progression-free survival (rPFS) and overall survival (OS). Subsequently, Kaplan-Meier curves were analyzed using Getdata 2.26 to extract the data pertaining to PFS, rPFS and OS at 6, 12, 18, 24, 30, and 36 months.

### Data analysis

To compare multiple treatments for progression-free survival (PFS), radiographic progression-free survival (rPFS) and overall survival (OS) at each time point, a network meta-analysis (NMA) was conducted using Stata 15.1 software (StataSE, United States). The NMA allowed for both direct and indirect comparisons between treatments. Odds ratios (OR) and 95% confidence intervals (CI) were calculated to evaluate the effects of treatments on PFS, rPFS and OS at each time point. Furthermore, treatment ranking was performed using the surface under the cumulative ranking curve (SUCRA) values. The significance of the effect size between any treatment pair was determined using the net-league table, also known as a matrix in algebra. Inconsistency tests and consistency tests were conducted to examine the presence of inconsistency in the results.

To generate Napierian logarithm odds ratios (lnOR) and standard errors of lnOR (selnOR) for each study, conventional meta-analyses were conducted using Stata 15.1 software. The resulting data, including lnOR and selnOR for OS, PFS and rPFS, were then input into Rstudio 4.1.2 and proceed the network meta-analysis (NMA).

If the I^2^ statistic was less than 50% and the *p*-value was greater than 0.01, a fixed-effect model was implemented. If the I^2^ statistic was between 50% and 75%, a random-effect model was applied. If the I^2^ statistic exceeded 75%, a Galbraith plot was used to identify and exclude any studies outside the outlined range. Markov-chain Monte Carlo (MCMC) simulations were utilized to obtain posterior distributions, with a burn-in of 20,000 iterations and 150,000 iterations of 4 each chain, with a thinning interval of 10 for each outcome. Brooks-Gelman-Rubin diagnostics and trace plots were employed to assess and visualize the convergence of the model over iterations. Matrices were also generated using Rstudio 4.1.2.

## Results

### Characteristics of the included studies

**FIGURE 1:**
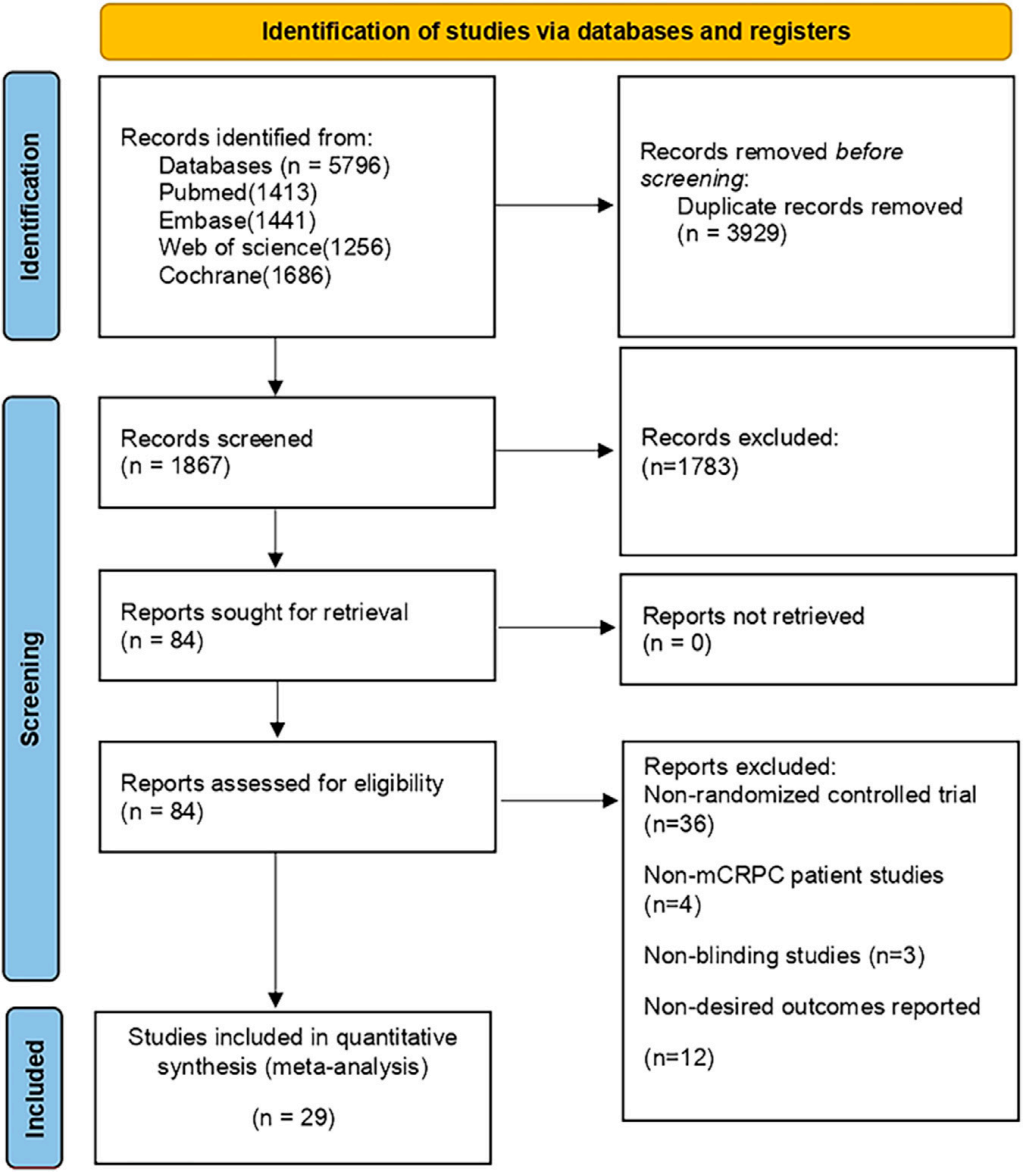
PRISMA flow diagram for the systematic review.

**FIGURE 2:**
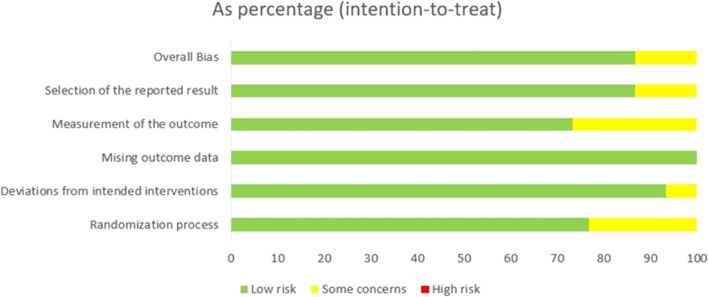
Risk of bias graph presented as percentage across all included studies.

During the initial search, a total of 5,796 publications were identified. After removing duplicates and screening titles and abstracts, 1,078 studies were considered eligible for full review. Eventually, 29 studies (Fizazi et al., 2012; Kluetz et al., 2013; Rathkopf et al., 2014; Fizazi et al., 2015; Ryan et al., 2015; Saad et al., 2015; Beer et al., 2017; Bouman-Wammes et al., 2018; Clarke et al., 2018; Miller et al., 2018; Monk et al., 2018; Corn et al., 2019; de Bono et al., 2019; De Wit et al., 2019; Armstrong et al., 2020; De Bono et al., 2020; Fizazi et al., 2020; Hussain et al., 2020; Annala et al., 2021; Hedley Carr et al., 2021; Madan et al., 2021; Saad et al., 2021; Sternberg et al., 2021; Sweeney et al., 2021; Crabb et al., 2022; Powles et al., 2022; Vogelzang et al., 2022; Fizazi et al., 2023) were included in our analysis, as depicted in Figure 1. Among these studies, there were 29 RCTs involving a total of 12,706 patients and investigating 16 different interventions. The interventions encompassed castratepre (Abiraterone or Enzalutamide + prednisone), prednisone, castratepreparp (Abiraterone or Enzalutamide + prednisone + olaparib), chempre (Docetaxel or Cabazitaxel + prednisone), chempre DCVAC (DCVAC + docetaxel + prednisone), castratepre400ipa (abiraterone + prednisone + 400 mg ipatasertib), chemprept (cabazitaxel or docetaxel + carboplatin + prednisone), chempretarget (capivasertib or cabozantinib + docetaxel + prednisone), chemprePD1 (ipilimumab + docetaxel + prednisone), castrateprepd1 (atezolizumab + enzalutamide + prednisone), target (cabozantinib), castratepre200ipa (abiraterone + prednisone + 200 mg ipatasertib), 2castratepre (Abiraterone and Enzalutamide), castratepretarget (ipatasertib or tivantinib + abiraterone + predisone) and parp (Olaparib or rucaparib) and targetpre (buparlisib or Orteronel + prednisone). A detailed description of the included studies can be found in Table 1. The reported median follow-up period ranged from 8.9 months to 54.8 months. The assessment of the risk of bias is presented in Figure 2.

**TABLE 1:** Characteristics of first-line systemic therapy for metastatic castration-resistant prostate cancer studies included in the network meta-analysis.

Author/year	Age	Treament	Drug type	Size	Study type	Follow-up (months)	Outcomes
Carr2021	NA	olaparib + abiraterone + predisone	castratepreparp	25	RCT	NA	rPFS
	NA	abiraterone + predisone	castratepre	21			
Clarke2018	70 (65–75)	olaparib + abiraterone + predisone	castratepreparp	71	RCT	15.9 (8.1–25.5)	OS,rPFS
	67 (62–74)	abiraterone + predisone	castratepre	71		24.5 (8.1–27.6)	
Crabb2022	NA	capivasertib + docetaxel + predisone	chempretarget	75	RCT	35	OS
	NA	docetaxel + predisone	chempre	75		32	
Sweeney2021	69 (47–93)	ipatasertib + abiraterone + predisone	castratepretarget	554	RCT	19 (0–33)	rPFS,PFS
	70 (44–90)	abiraterone + predisone	castratepre	547			
	66 (48–91)	buparlisib + predisone	targetpre	17			
Bono20191	68.8 (7.2)	abiraterone + predisone+200mgipatasertib	castratepre200ipa	86	RCT	NA	OS,PFS,rPFS
	67.6 (7.8)	abiraterone + predisone	castratepre	83			
	66.9 (8.5)	abiraterone + predisone+400mgipatasertib	castratepre400ipa	84			
Madan2020	69 (54–80)	cabozantinib + docetaxel + Prednisone	chempretarget	13	RCT	NA	OS
	69 (50–83)	docetaxel + prednisone	chempre	12			
Monk2018	67 (43–84)	tivantinib + abiraterone + predisone	castratepretarget	52	RCT	8.9 (2.3–19.6)	PFS
	66.5 (48–85)	abiraterone + predisone	castratepre	26			
Powles2022	NA	atezolizumab + enzalutamide + prednisone	castrateprepd1	380	RCT	NA	OS,PFS,rPFS
	NA	enzalutamide + prednisone	castratepre	379			
Voge2022	68 (46–89)	DCVAC + docetaxel + predisone	chempreDCVAC	787	RCT	NA	OS
	69 (46–89)	docetaxel + predisone	chempre	395			
Annala2020	67.5 (60.3–71.0)	abiraterone + predisone	castratepre	50	RCT	NA	OS
	68.0 (59.0–73.0)	Cabazitaxel + predisone	chempre	45			
Saad2021	71 (66–78)	apalutamide + abiraterone + predisone	2castratepre	492	RCT	54·8 (51·5–58·4)	OS,Rpfs
	71 (65–77)	abiraterone + predisone	castratepre	490			
	67.7 (7.75)	prednisone	prednisone	71			
	69 (49–86)	enzalutamide + predisone	castratepre	64			
Bono2019	70.0 (46–85)	enzalutamide + prednisone	castratepre	129	RCT	9.2	OS,PFS,rPFS
	71.0 (45–88)	cabazitaxel + predisone	chempre	126			
Bono2020	69 (47–91)	Olaparib	parp	256	RCT	13.2	OS,Rpfs
	69 (49–87)	abiraterone + predisone	castratepre	131			
Fizazi2020	69.0 (63.0–74.0)	lpilimumab + docetaxel + predisone	chemprePD1	399	RCT	50 (40.7, 72.0)	OS
	67.5 (62.0–72.5)	docetaxel + predisone	chempre	400			
Fizazi2023	70 (45–90)	rucaparib	parp	270	RCT	NA	rPFS,PFS
	71 (47–92)	abiraterone + predisone	castratepre	135			
Hussain2020	NA	abiraterone + predisone	castratepre	162	RCT	21.9	OS,PFS
	NA	Olaparib	parp	83			
Sterberg2021	74 (70–88)	abiraterone + predisone	castratepre	69	RCT	9.2	OS,PFS,rPFS
	76 (70–85)	Cabazitaxel + predisone	chempre	66			
Bouman2018	69.2 (61–85)	docetaxel + predisone + carboplatin	chemprept	36	RCT	NA	OS,PFS
	70.1 (60–84)	docetaxel + predisone	chempre	37			
Corn2019	72 (67–76)	cabazitaxel + carboplatin + predisone	chemprept	81	RCT	31·0 (20·5–37·1)	OS,PFS
	66 (61–69)	cabazitaxel + carboplatin	chempre	79			
Fizazi2015	69.5 (43–89)	Orteronel + predisone	targetpre	734	RCT	10.6 (0.2–29.5)	OS,PFS
	70 (48–87)	prednisone	prednisone	365			
Kluetz2013	NA	abiraterone + predisone	castratepre	546	RCT	NA	OS
	NA	prednisone	prednisone	542			
Smith2016	69.5 (35–87)	cabozantinib	target	682	RCT	NA	OS,Rpfs
	69 (43–89)	prednisone	prednisone	346			
Fizazi2012	69 (42–95)	abiraterone + predisone	castratepre	797	RCT	20·2 (18·4–22·1)	OS,PFS,rPFS
	69 (39–90)	prednisone	prednisone	398			
Beer20171	72 (43–93)	enzalutamide + prednisone	castratepre	872	RCT	NA	rPFS,PFS
	71 (42–93)	prednisone	prednisone	845			
Miller2018	70 (64–76.5)	abiraterone + predisone	castratepre	264	RCT	NA	OS,Rpfs
	71 (64–77)	prednisone	prednisone	824			
Rathkopf2014	71 (65–77)	abiraterone + predisone	castratepre	546	RCT	27.1	OS,PFS,rPFS
	70 (63–76)	prednisone	prednisone	542			
Ryan2015	NA	abiraterone + predisone	castratepre	546	RCT	49·2 (47·0–51·8)	OS
	NA	prednisone	prednisone	542			
Armstrong2020	NA	enzalutamide + prednisone	castratepre	872	RCT	69	OS
	NA	prednisone	predisone	845			
Saad2015	71·0 (65·0–77·0)	Orteronel + prednisone	targetpre	781	RCT	20.7 (14·2–25·4)	OS,Rpfs
	72·0 (66·0–77·0)	prednisone	prednisone	779			

OS: overall survival; PFS: progression free survival; rPFS: radiographic progression-free survival; RCT: randomized controlled trials.

### OS at each time point

**FIGURE 3:**
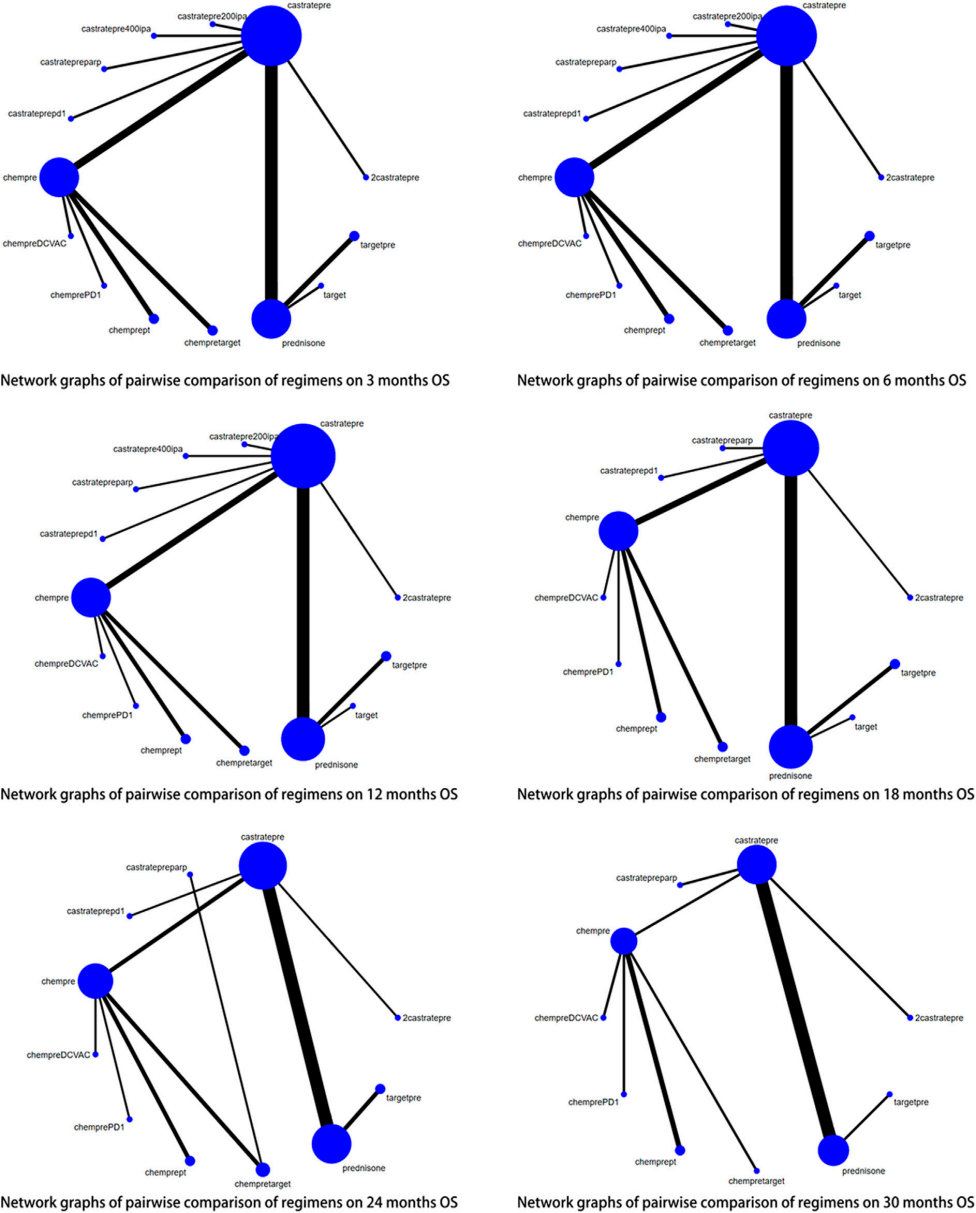
Network graphs of pairwise comparison of regimens on each time point of the overall survival; OS, overall survival; castratepre (Abiraterone or Enzalutamide + prednisone), prednisone, castratepreparp (Abiraterone or Enzalutamide + prednisone + olaparib), chempre (Docetaxel or Cabazitaxel + prednisone), chempre DCVAC (DCVAC + docetaxel + prednisone), castratepre400ipa (abiraterone + prednisone + 400 mg ipatasertib), chemprept (cabazitaxel or docetaxel + carboplatin + prednisone), chempretarget (capivasertib or cabozantinib + docetaxel + prednisone), chemprePD1 (ipilimumab + docetaxel + prednisone), castrateprepd1 (atezolizumab + enzalutamide + prednisone), target (cabozantinib), castratepre200ipa (abiraterone + prednisone + 200 mg ipatasertib), 2castratepre (Abiraterone and Enzalutamide), and targetpre (buparlisib or Orteronel + prednisone).

Among the 29 included articles, 18 provided data on overall survival (OS) outcomes. For this study, sufficient data were available at 3, 6, 12, 24, and 30 months to conduct the network meta-analysis (NMA). The pairwise comparison of treatment regimens for each OS time point is presented in Figure 3. Castratepre (Abiraterone or Enzalutamide + prednisone) was the most commonly used intervention, and chempre (Docetaxel or Cabazitaxel + prednisone) was the most frequently compared treatment. Considering the widespread clinical use of castratepre as the first-line standard treatment, it was chosen as the primary reference, while the intervention with the highest SUCRA ranking served as the secondary reference. Detailed results of direct and indirect comparisons of 16 interventions at each time point are shown in the Supplementary Table S1A–F.

At the 3^rd^ month, castratepre400ipa showed significant superiority over castratepre (OR = 1.93, 95%CI: 1.02–3.63) and prednisone (OR = 2.53, 95%CI: 1.22–5.24), indicating its greater significance in the initial 3 months.

At the 6^th^ month, no treatment demonstrated a significant advantage over castratepre.

At the 12^th^ month, chempre (OR = 1.66, 95%CI: 1.08–2.58), chempretarget (OR = 2.88, 95%CI: 1.09–7.62), and chemprePD1 (OR = 2.14, 95%CI: 1.13–4.05) exhibited a significant increase in OS compared to castratepre. According to the SUCRA rankings, chempretarget ranked first, followed by chemprePD1.

At the 18^th^ month, chempre (OR = 2.63, 95%CI: 1.71–4.04), chempreDCVAC (OR = 2.03, 95%CI: 1.16–3.56), chemprept (OR = 3.1, 95%CI: 1.54–6.22), chempretarget (OR = 5.43, 95%CI: 2.44–12.09), and chemprePD1 (OR = 3.82, 95%CI: 2.13–6.87) exhibited a significant increase in the OS rate compared to castratepre. According to the SUCRA rankings, chempretarget ranked first, followed by chemprePD1.

At the 24^th^ month, castratepreparp (OR = 6.12, 95%CI: 2.14–17.52), chempre (OR = 3.04, 95%CI: 1.77–5.2), chempreDCVAC (OR = 2.74, 95%CI: 1.51–4.96), chemprept (OR = 2.89, 95%CI: 1.35–6.19), chempretarget (OR = 5.46, 95%CI: 2.41–12.35), chemprePD1 (OR = 4.91, 95%CI: 2.57–9.4), and castrateprepd1 (OR = 1.64, 95%CI: 1.16–2.32) showed a significant increase in the OS rate compared to castratepre. Castratepreparp ranked the highest in terms of SUCRA, while chempretarget ranked second.

At the 30^th^ month, chempre (OR = 2.44, 95%CI: 1.05–5.66), chempreDCVAC (OR = 2.59, 95%CI: 1.08–6.22), chempretarget (OR = 5.14, 95%CI: 1.75–15.1), and chemprePD1 (OR = 3.93, 95%CI: 1.55–9.97) exhibited a significantly higher OS rate compared to castratepre. According to the SUCRA rankings, chempretarget achieved the best performance, followed by chemprePD1.

Regarding overall survival (OS), significant differences were observed between castratepre and chempre, chempretarget, chemprePD1, as well as chempreDCVAC from 12 to 30 months. Additionally, chempre, chempretarget, and chemprePD1 showed significant differences from 12 to 30 months, while chempreDCVAC exhibited significance from 18 to 30 months Table 2 provides a comprehensive summary of interventions with significant results compared to castratepre.

**TABLE 2:** Overall survival for each time point for interventions that were significant compared to castratepre (shown as odds ratio and 95% confidence intervals).

Time point	Control group	castratepreparp	chempre	chempreDCVAC	castratepre400ipa	chemprept	chempretarget	chemprePD1	castrateprepd1	Target	castratepre200ipa	2castratepre	Targetpre
3 months	castratepre	×	×	×	1.93 (1.02; 3.63)	×	×	×	×	-	×	×	-
	prednisone	×	×	×	2.53 (1.22; 5.24)	×	×	×	×	×	×	×	×
6 months	castratepre	×	×	×	×	×	×	×	×	-	×	×	-
	prednisone	√	√	×	√	×	×	×	×	×	×	√	×
12 months	castratepre	×	1.66 (1.08; 2.58)	×	×	×	2.88 (1.09; 7.62)	2.14 (1.13; 4.05)	×	-	×	×	-
	prednisone	×	√	√	√	√	√	√	×	×	×	√	×
18 months	castratepre	×	2.63 (1.71; 4.04)	2.03 (1.16; 3.56)	-	3.10 (1.54; 6.22)	5.43 (2.44; 12.09)	3.82 (2.13; 6.87)	×	-	-	×	-
	prednisone	×	√	√	-	√	√	√	×	×	-	√	×
24 months	castratepre	6.12 (2.14; 17.52)	3.04 (1.77; 5.20)	2.74 (1.51; 4.96)	-	2.89 (1.35; 6.19)	5.46 (2.41; 12.35)	4.91 (2.57; 9.40)	1.64 (1.16; 2.32)	-	-	×	-
	prednisone	√	√	√	-	√	√	√	√	×	-	√	×
30 months	castratepre	×	2.44 (1.05; 5.66)	2.59 (1.08; 6.22)	-	×	5.14 (1.75; 15.10)	3.93 (1.55; 9.97)	-	-	-	×	-
	prednisone	√	√	√	-	√	√	√	-	-	-	√	×

Castratepre (Abiraterone or Enzalutamide + prednisone), prednisone, castratepreparp (Abiraterone or Enzalutamide + prednisone + olaparib), chempre (Docetaxel or Cabazitaxel + prednisone), chempre DCVAC (DCVAC + docetaxel + prednisone), castratepre400ipa (abiraterone + prednisone + 400 mg ipatasertib), chemprept (cabazitaxel or docetaxel + carboplatin + prednisone), chempretarget (capivasertib or cabozantinib + docetaxel + prednisone), chemprePD1 (ipilimumab + docetaxel + prednisone), castrateprepd1 (atezolizumab + enzalutamide + prednisone), target (cabozantinib), castratepre200ipa (abiraterone + prednisone + 200 mg ipatasertib), 2castratepre (Abiraterone and Enzalutamide), and targetpre (buparlisib or Orteronel + prednisone).

√: the treatment on the top is significant compared to the control group on the left; ×: the treatment on the top is not significant compared to the Control group on the left.

### PFS at each time point

**FIGURE 4:**
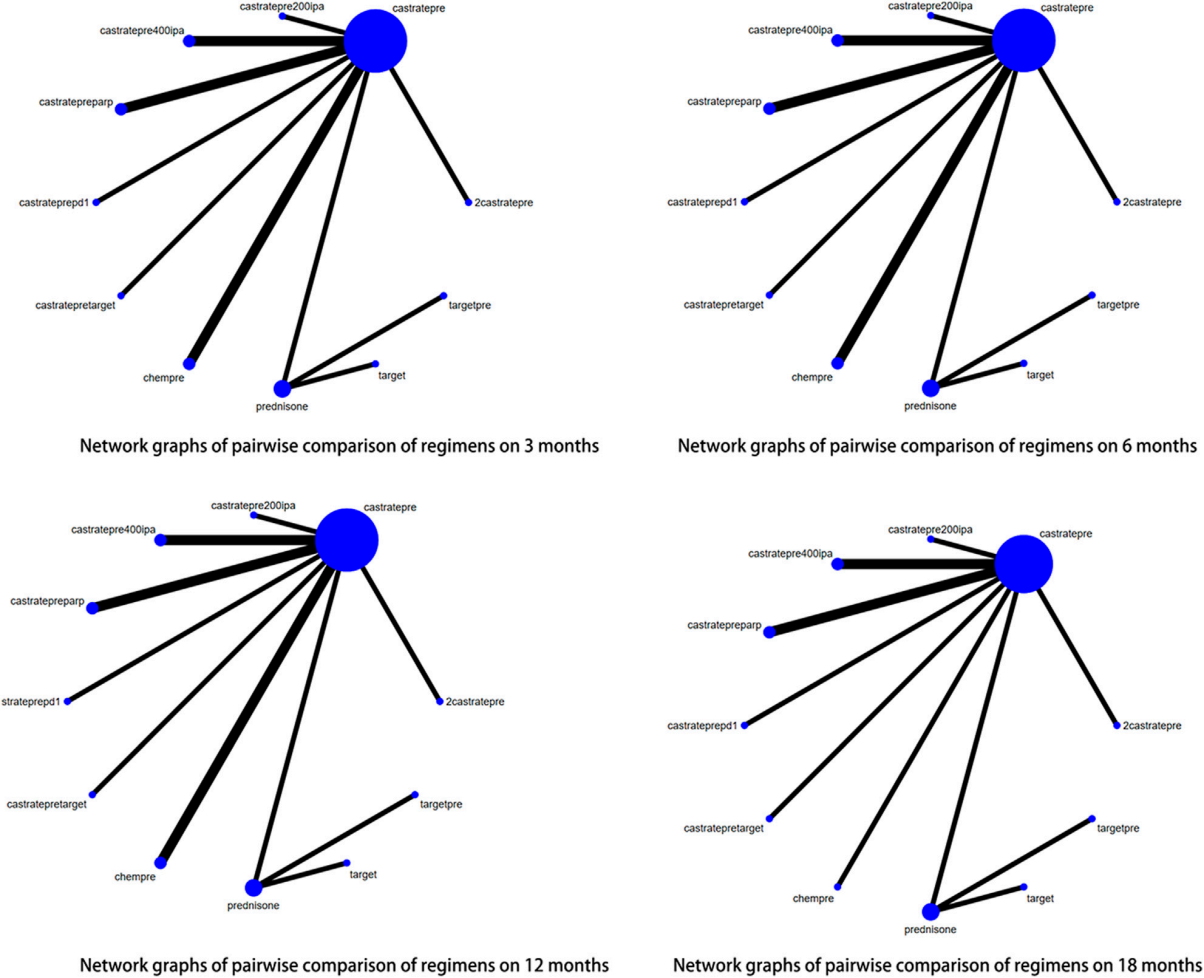
Network graphs of pairwise comparison of regimens on each time point of the Progression free survival; PFS, Progression free survival; castratepre (Abiraterone or Enzalutamide + prednisone), prednisone, chemprept (cabazitaxel or docetaxel + carboplatin + prednisone), chempre (Docetaxel or Cabazitaxel + prednisone), castratepretarget (ipatasertib or tivantinib + abiraterone + predisone), parp (Olaparib or rucaparib), targetpre (buparlisib or Orteronel + prednisone).

For progression-free survival (PFS), outcomes from 14 out of 29 articles were reported. Adequate data were available at 4 time points, namely 3, 6, 12, and 18 months, to conduct a network meta-analysis (NMA) for PFS. Figure 4 displays network graphs illustrating the pairwise comparison of regimens at each time point for PFS. Castratepre was the most frequently used intervention, and the most common comparisons were between chempre and castratepre, as well as between castratepre and castratepretarget. A detailed comparison of 16 interventions at each time point is presented in Supplementary Tables S2A–D.

At the 3^rd^ month, there was a significant increase in PFS rates with chemprept (OR = 8.68, 95% CI: 4.09–18.4), chempre (OR = 3.92, 95% CI: 2.58–5.98), castratepretarget (OR = 2.14, 95% CI: 1.21–3.79), and parp (OR = 1.96, 95% CI: 1.09–3.52) compared to castratepre. Among these interventions, chemprept ranked the highest according to the SUCRA rankings, followed by chempre.

At the 6^th^ month, there was a significant improvement in PFS compared to castratepre with chemprept (OR = 5.73, 95% CI: 2.54–12.92), chempre (OR = 2.97, 95% CI: 1.73–5.09), castratepretarget (OR = 2.86, 95% CI: 1.36–6), and parp (OR = 1.96, 95% CI: 1.09–3.52). When compared to the top-ranked intervention chemprept, chempre ranked second.

At the 12^th^ month, there was a significant increase in PFS for chempre (OR = 4.21, 95% CI: 1.16–15.28), castratepretarget (OR = 2.82, 95% CI: 1.14–6.95), and parp (OR = 3.14, 95% CI: 1.86–5.32) compared to castratepre. Among these interventions, chempre had the highest SUCRA ranking, followed by parp.

At the 18^th^ month, there was a significant increase in PFS for parp (OR = 3.08, 95% CI: 1.67–5.71) compared to castratepre.

Regarding progression-free survival (PFS), significant effects were observed for interventions ranging from 3 to 18 months, including chemprept, chempre, castratepretarget, and parp in descending order of their impact. Table 3 provides a comprehensive summary of the interventions with significant outcomes when compared to castratepre.

**TABLE 3:** Progression free survival for each time point for interventions that were significant compared to castratepre (shown as odds ratio and 95% confidence intervals).

Time point	Control group	chemprept	Chempre	castratepretarget	Parp	Targetpre
3 months	castratepre	8.68 (4.09; 18.40)	3.92 (2.58; 5.98)	2.14 (1.21; 3.79)	1.96 (1.09; 3.52)	-
	prednisone	√	√	√	√	1.52 (1.10; 2.09)
6 months	castratepre	5.73 (2.54; 12.92)	2.97 (1.73; 5.09)	2.86 (1.36; 6.00)	2.34 (1.36; 4.02)	-
	prednisone	√	√	√	√	1.52 (1.00; 2.30)
12 months	castratepre	×	4.21 (1.16; 15.28)	2.82 (1.14; 6.95)	3.14 (1.86; 5.32)	-
	prednisone	√	√	√	√	1.73 (1.29; 2.31)
18 months	castratepre	×	×	×	3.08 (1.67; 5.71)	-
	prednisone	√	√	√	√	1.45 (1.00; 2.09)

Castratepre (Abiraterone or Enzalutamide + prednisone), prednisone, chemprept (cabazitaxel or docetaxel + carboplatin + prednisone), chempre (Docetaxel or Cabazitaxel + prednisone), castratepretarget(ipatasertib or tivantinib + abiraterone + predisone), parp (Olaparib or rucaparib), targetpre (buparlisib or Orteronel + prednisone).

√: the treatment on the top is significant compared to the control group on the left; ×: the treatment on the top is not significant compared to the Control group on the left.

### rPFS at each time point

**FIGURE 5:**
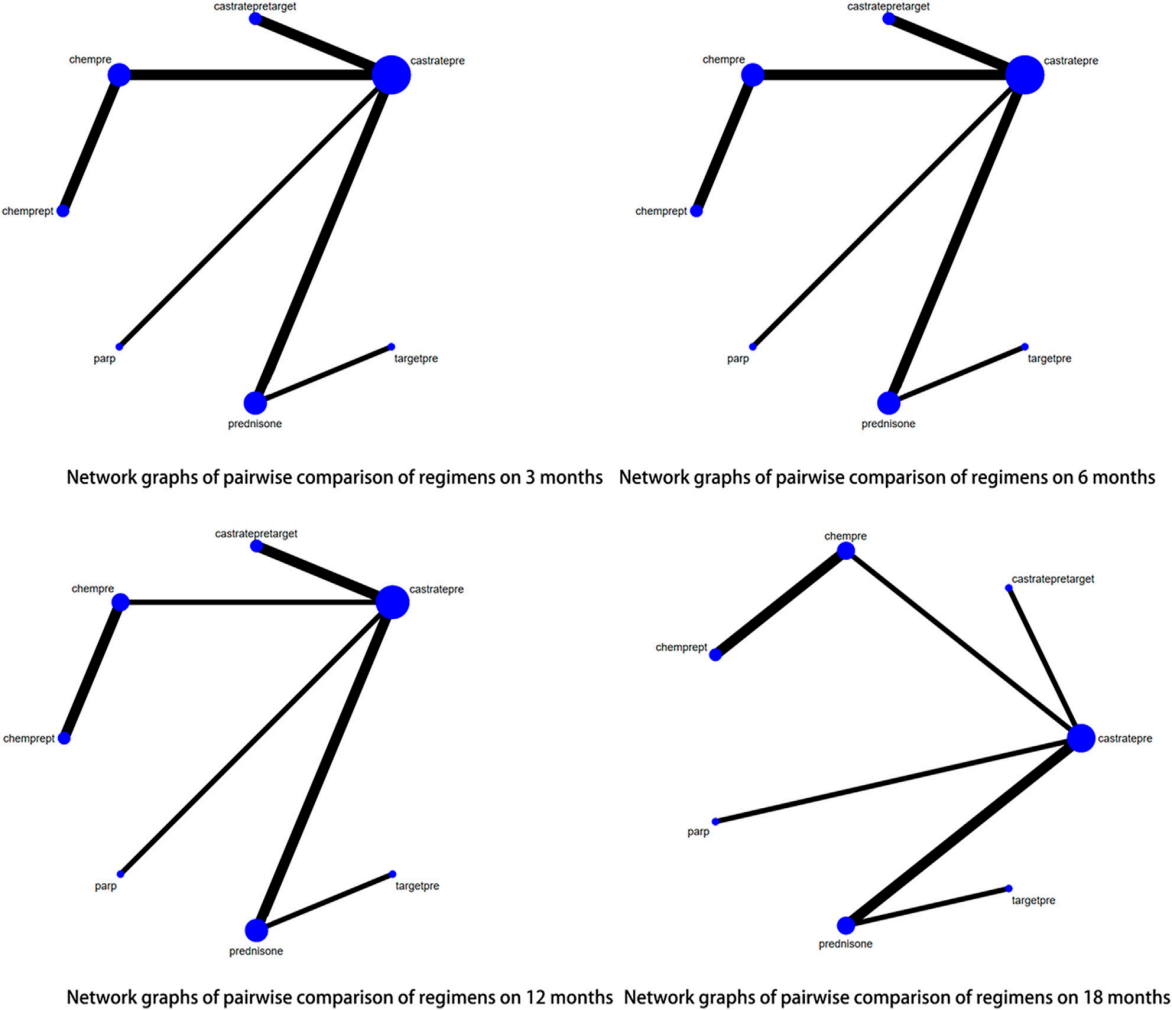
Network graphs of pairwise comparison of regimens on each time point of the radiographic Progression free survival; rPFS: radiographic Progression free survival; castratepre (Abiraterone or Enzalutamide + prednisone), prednisone, target (cabozantinib), castratepretarget (ipatasertib or tivantinib + abiraterone + predisone), chempre (Docetaxel or Cabazitaxel + prednisone), castratepreparp (Abiraterone or Enzalutamide + prednisone + olaparib), targetpre (buparlisib or Orteronel + prednisone), 2castratepre (Abiraterone and Enzalutamide), castratepre400ipa (abiraterone + prednisone + 400 mg ipatasertib), castrateprepd1 (atezolizumab + enzalutamide + prednisone), castratepre200ipa (abiraterone + prednisone + 200 mg ipatasertib).

Out of the 29 articles included in this study, 16 reported outcomes related to radiographic progression-free survival (rPFS). Sufficient data were available at 3, 6, 12, and 18 months to perform a network meta-analysis (NMA) for rPFS. The pairwise comparison of different treatment regimens at each rPFS time point is illustrated in Figure 5. Among the agents studied, castratepre was the most frequently utilized, and comparisons were predominantly made between castratepreparp and chempre. A detailed comparison of 17 interventions at each time point is presented in Supplementary Tables 3A–D.

At the 3^rd^ month, there was a statistically significant increase in radiographic progression-free survival (rPFS) rates observed with the following interventions: target (OR = 6.88, 95% CI: 1.6–29.6), castratepretarget (OR = 4.15, 95% CI: 1.53–11.2), chempre (OR = 2.99, 95% CI: 1.91–4.68), and castratepreparp (OR = 2.54, 95% CI: 1.14–5.63) compared to castratepre. Among these interventions, target achieved the highest ranking according to the SUCRA rankings, followed by castratepretarget in the second position.

At the 6^th^ month, there was a statistically significant improvement in radiographic progression-free survival (rPFS) compared to castratepre with the following interventions: chempre (OR = 2.57, 95% CI: 1.71–3.86), castratepreparp (OR = 2.25, 95% CI: 1.16–4.38), 2castratepre (OR = 1.62, 95% CI: 1.15–2.3), and castratepre400ipa (OR = 1.73, 95% CI: 1.18–2.53). In terms of ranking according to the SUCRA scores, chempre held the highest position, followed by castratepreparp in the second rank.

At the 12^th^ month, there was a statistically significant increase in radiographic progression-free survival (rPFS) for chempre (OR = 3.74, 95% CI: 2.21–6.59), castratepreparp (OR = 1.98, 95% CI: 1.1–3.56), 2castratepre (OR = 1.45, 95% CI: 1.11–1.89), and castratepre400ipa (OR = 1.71, 95% CI: 1.24–2.36) compared to castratepre. Among these interventions, chempre achieved the highest SUCRA ranking, followed by castratepreparp.

At the 18^th^ month, there was a statistically significant increase in radiographic progression-free survival (rPFS) for chempre (OR = 2.94, 95% CI: 1.19–7.26), 2castratepre (OR = 1.52, 95% CI: 1.18–1.95), castratepre400ipa (OR = 1.58, 95% CI: 1.15–2.17), and castrateprepd1 (OR = 2.64, 95% CI: 1.09–6.4) compared to castratepre.

In terms of radiographic progression-free survival (rPFS), the interventions that showed a significant impact compared to castratepre spanned from 3 to 18 months. These interventions, in descending order of their effectiveness, included chempre, castratepreparp, 2castratepre, and castratepre400ipa. A comprehensive summary of the interventions with significant outcomes compared to castratepre can be found in Table 4.

**TABLE 4:** Radiographic Progression free survival for each time point for interventions that were significant compared to castratepre (shown as odds ratio and 95% confidence intervals).

Time point	Control group	Target	castratepretarget	chempre	castratepreparp	Targetpre	2castratepre	castratepre400ipa	castrateprepd1	castratepre200ipa
3 months	castratepre	6.88 (1.60; 29.64)	4.15 (1.53; 11.25)	2.99 (1.91; 4.68)	2.54 (1.14; 5.63)	×	×	×	×	×
	prednisone	√	√	√	√	√	×	-	-	-
6 months	castratepre	-	×	2.57 (1.71; 3.86)	2.25 (1.16; 4.38)	-	1.62 (1.15; 2.30)	1.73 (1.18; 2.53)	×	×
	prednisone	3.38 (2.45; 4.65)	√	√	√	√	√	√	√	√
12 months	castratepre	-	×	3.74 (2.12; 6.59)	1.98 (1.10; 3.56)	-	1.45 (1.11; 1.89)	1.71 (1.24; 2.36)	×	×
	prednisone	2.01 (1.23; 3.28)	√	√	√	√	√	√	√	√
18 months	castratepre	-	×	2.94 (1.19; 7.26)	×	×	1.52 (1.18; 1.95)	1.58 (1.15; 2.17)	2.64 (1.09; 6.40)	×
	prednisone	×	√	√	√	√	√	√	√	×

Castratepre (Abiraterone or Enzalutamide + prednisone), prednisone, target (cabozantinib), castratepretarget(ipatasertib or tivantinib + abiraterone + predisone), chempre (Docetaxel or Cabazitaxel + prednisone), castratepreparp (Abiraterone or Enzalutamide + prednisone + olaparib), targetpre (buparlisib or Orteronel + prednisone), 2castratepre (Abiraterone and Enzalutamide), castratepre400ipa (abiraterone + prednisone + 400 mg ipatasertib), castrateprepd1 (atezolizumab + enzalutamide + prednisone), castratepre200ipa (abiraterone + prednisone + 200 mg ipatasertib).

√: the treatment on the top is significant compared to the control group on the left; ×: the treatment on the top is not significant compared to the Control group on the left.

The Brooks-Gelman-Rubin diagnostic indicated that the inferential iterations for each Markov-chain Monte Carlo (MCMC) were stable and reproducible across all outcomes. Furthermore, the convergence of the model for all outcomes was confirmed using the history feature. Supplementary Figures 1A–C and Supplementary Figures 2A–C could provide comprehensive details of the results.

### Heterogeneity

For all outcomes, the Brooks-Gelman-Rubin diagnostic indicated that the inferential iterations for each Markov-chain Monte Carlo (MCMC) were stable and reproducible. Additionally, we employed the history feature to verify the convergence of the model for all outcomes. Comprehensive results can be found in Supplementary Figures 1A–C and Supplementary Figures 2A–C, providing further details.

The heterogeneity of the results in our study was all less than 30%, which demonstrated the robustness of our findings. Therefore, we did not conduct subgroup analysis and meta-regression to identify the source of heterogeneity.

## Discussion

The present study aimed to evaluate the efficacy of multiple regimens for the treatment of metastatic castration-resistant prostate cancer (mCRPC) through a comprehensive network meta-analysis (NMA) of RCTs.

To our knowledge, this is the first comparison of various treatment regimens alone or in combination at each time point for mCRPC patients with overall survival (OS), progression-free survival (PFS), and radiographic progression-free survival (rPFS). The results of the study are as follows: In terms of OS, compared with the mainstream castratepre, we found that chempre, chempretarget, and chemprePD1 showed a significant survival advantage from the 12th month, and chemprept and chempreDCVAC also demonstrated better efficacy from the 18th month. However, due to the limited availability of data, we could not obtain data beyond 30 months. We believe that chempretarget is the preferred choice for improving patients’ OS. In terms of PFS from 3 to 18 months, PARP inhibitors showed a clear advantage and, therefore, are the preferred choice for improving PFS in patients. Chempre demonstrated better efficacy in rPFS from 3 to 18 months and, thus, is considered the preferred treatment for improving rPFS.

Chempretarget showed encouraging results for overall survival. The studies included Capivasertib + Docetaxel and Cabozantinib + Docetaxel. Capivasertib is a potent selective inhibitor of three AKT subtypes (AKT1/2/3) and has demonstrated efficacy in various cancers (Coleman et al., 2021; Gasmi et al., 2021). The PI3K/AKT/PTEN pathway has been shown to be abnormally activated in patients resistant to taxane chemotherapy (Liu et al., 2015), which may explain why Capivasertib + Docetaxel works better than first-line chemotherapy treatment. It is not yet known why adding Capivasertib to chemotherapy improves OS, and the ongoing Phase 3 phase 3 CAPItello-280 trial (NCT05348577) may provide us with the answer. Cabozantinib targets VEGFR2 and C-MET and is used in various cancers (Grüllich, 2018). The combination with docetaxel allows for a lower dose of cabozantinib over a longer period, leading to sustained clinical benefits. Recent pharmacokinetic and pharmacodynamic studies have shown that combining a standard dose of docetaxel with approximately 20 mg of cabozantinib per day can optimize anti-tumor effects and potential treatment duration (Chen et al., 2018). In addition, biological compensation mechanisms caused by the discontinuation of antiangiogenic drugs such as codo can improve clinical efficacy by limiting clinical rebound through cytotoxic therapy such as docetaxel (Smith et al., 2016; Zhang et al., 2017).

For PFS, our study demonstrates that PARP inhibitors seem to have better efficacy. The PARP inhibitors included in our analysis were rucaparib and olaparib, respectively. Approximately 30% of mCRPC patients have DNA gene damage (Abida et al., 2017; Freedland and Aronson, 2017), with BRCA1 and BRCA2 being genes involved in homologous repair (Walsh, 2015; Blackford and Jackson, 2017). PARP inhibitors mainly induce DNA double-strand breaks and exploit homologous recombination repair defects associated with these pathological genes through PARP trapping (O'Connor, 2015). The efficacy of PARP inhibitors in the progression-free survival of mCRPC patients has been widely reported (Mateo et al., 2020), with the greatest benefit observed in the BRCA subgroup. The study found that the median duration of olaparib exposure was shorter in patients who transitioned from control treatment to olaparib (4.8 months) than in patients randomly assigned to receive olaparib (7.6 months). Therefore, early treatment with olaparib may have advantages over use later in the course of the disease (Hussain et al., 2020). Although the studies we included had substantial crossover from control treatment to parp, improvements in patients’ PFS were noted. Recently Several phase 2-3 studies have demonstrated the clinical efficacy of combining PARP inhibitors with second-generation ARPI as a frontline treatment for metastatic castration-resistant prostate cancer patients. These studies suggest that the benefits of this combination therapy are particularly enhanced in patients with gene alterations associated with DNA damage, highlighting the potential advantages of this approach (Clarke et al., 2018; Hussain et al., 2020; Saad et al., 2021). However, limitations of trials include the immaturity of overall survival data and exploratory nature of some subgroup analyses.

Generally, ARPI (Androgen Receptor Pathway Inhibitors) is the preferred treatment option for mCRPC (metastatic castration-resistant prostate cancer) patients, given its well-established survival benefits and tolerability (Ryan et al., 2015; Beer et al., 2017; Annala et al., 2018). However, certain patients with adverse prognostic clinical features do not derive equivalent levels of benefit from ARPI therapy.Chemotherapy as a first-line treatment can overcome resistance mechanisms to androgen-targeted inhibitors, such as increased androgen signaling and PTEN loss (Fitzpatrick and de Wit, 2014; Antonarakis et al., 2015; Palapattu, 2016; Rescigno et al., 2018). The CARD trial demonstrated an overall survival advantage of cabazitaxel over ARPI. However, it is important to note that the population in the CARD trial had previously received docetaxel and ARPI treatment. Thus, the study was evaluating cabazitaxel as a third-line therapy in patients who were already known to have ARPI resistance in first and second-line treatments.

## Strengths and limitations

We conducted a comprehensive analysis by evaluating 16 first-line interventions using 29 carefully selected high-quality studies. The analysis covered a follow-up period of up to 30 months for overall survival (OS) and 18 months for progression-free survival (PFS) and radiographic progression-free survival (rPFS). We demonstrated the stability and replicability of each MCMC chain iteration using Brooks-Gelman-Rubin diagnostics and estimated the convergence of the model.

Despite the valuable insights provided by this network meta-analysis (NMA), several limitations need to be acknowledged. Firstly, although we compared various treatment combinations directly or indirectly, it is essential to recognize that this approach cannot fully replace head-to-head comparative clinical trials. Moreover, we have merely demonstrated an association between treatment and outcomes, without establishing a causal relationship. Therefore, direct comparative trials remain indispensable. Secondly, the quality of the trials included in this analysis may have been influenced by various types of bias, potentially impacting the overall validity of the outcomes. Thirdly, the study population consisted exclusively of patients with metastatic prostate cancer. Additionally, certain confounding factors (e.g., drug dose, number of focal metastases, patient risk class, etc.) had missing data in some trials, and we were unable to account for these factors through meta-regression. Therefore, caution should be exercised when interpreting the results of this NMA in light of these limitations.

## Conclusion

Chempretarget and PARP inhibitors demonstrate superior efficacy in improving survival outcomes for mCRPC patients compared to first-line treatment. However, further head-to-head comparisons are required to validate these findings.

## Data Availability

The original contributions presented in the study are included in the article/Supplementary Material, further inquiries can be directed to the corresponding author.

## References

[B1] Abida W. Armenia J. Gopalan A. Brennan R. Walsh M. Barron D. (2017). Prospective genomic profiling of prostate cancer across disease States reveals germline and somatic alterations that may affect clinical decision making. JCO Precis. Oncol. 2017, 1–16. eng. Epub 2017/08/22. 10.1200/po.17.00029 PMC5558263 28825054

[B2] Annala M. Fu S. Bacon J. V. W. Sipola J. Iqbal N. Ferrario C. (2021). Cabazitaxel versus abiraterone or enzalutamide in poor prognosis metastatic castration-resistant prostate cancer: a multicentre, randomised, open-label, phase II trial. Ann. Oncol. 32 (7), 896–905. 10.1016/j.annonc.2021.03.205 33836265

[B3] Annala M. Vandekerkhove G. Khalaf D. Taavitsainen S. Beja K. Warner E. W. (2018). Circulating tumor DNA genomics correlate with resistance to abiraterone and enzalutamide in prostate cancer. Cancer Discov. 8 (4), 444–457. 10.1158/2159-8290.Cd-17-0937 29367197

[B4] Antonarakis E. S. Lu C. Luber B. Wang H. Chen Y. Nakazawa M. (2015). Androgen receptor splice variant 7 and efficacy of taxane chemotherapy in patients with metastatic castration-resistant prostate cancer. JAMA Oncol. 1 (5), 582–591. eng. Epub 2015/07/17. 10.1001/jamaoncol.2015.1341 26181238 PMC4537351

[B5] Armstrong A. J. Lin P. Tombal B. Saad F. Higano C. S. Joshua A. M. (2020). Five-year survival prediction and safety outcomes with enzalutamide in men with chemotherapy-naïve metastatic castration-resistant prostate cancer from the PREVAIL trial. Eur. Urol. 78 (3), 347–357. eng. Epub 2020/06/13. 10.1016/j.eururo.2020.04.061 32527692

[B6] Beer T. M. Armstrong A. J. Rathkopf D. Loriot Y. Sternberg C. N. Higano C. S. (2017). Enzalutamide in men with chemotherapy-naïve metastatic castration-resistant prostate cancer: extended analysis of the phase 3 PREVAIL study. Eur. Urol. 71 (2), 151–154. eng. Epub 2016/08/02. 10.1016/j.eururo.2016.07.032 27477525 PMC5570461

[B7] Bieńkowski M. Tomasik B. Braun M. Jassem J. (2022). PARP inhibitors for metastatic castration-resistant prostate cancer: biological rationale and current evidence. Cancer Treat. Rev. 104, 102359. Cited in: Pubmed; PMID 35190335. 10.1016/j.ctrv.2022.102359 35190335

[B8] Blackford A. N. Jackson S. P. (2017). ATM, ATR, and DNA-PK: the trinity at the heart of the DNA damage response. Mol. Cell 66 (6), 801–817. eng. Epub 2017/06/18. 10.1016/j.molcel.2017.05.015 28622525

[B9] Bouman-Wammes E. W. van den Berg H. P. de Munck L. Beeker A. Smorenburg C. H. Vervenne W. L. (2018). A randomised phase II trial of docetaxel versus docetaxel plus carboplatin in patients with castration-resistant prostate cancer who have progressed after response to prior docetaxel chemotherapy: the RECARDO trial. Eur. J. Cancer 90, 1–9. 10.1016/j.ejca.2017.11.021 29268139

[B10] Chen W. Chen R. Li J. Fu Y. Yang L. Su H. (2018). Pharmacokinetic/pharmacodynamic modeling of schedule-dependent interaction between docetaxel and cabozantinib in human prostate cancer xenograft models. J. Pharmacol. Exp. Ther. 364 (1), 13–25. eng. Epub 2017/11/01. 10.1124/jpet.117.243931 29084815

[B11] Clarke N. Wiechno P. Alekseev B. Sala N. Jones R. Kocak I. (2018). Olaparib combined with abiraterone in patients with metastatic castration-resistant prostate cancer: a randomised, double-blind, placebo-controlled, phase 2 trial. Lancet Oncol. 19 (7), 975–986. 10.1016/S1470-2045(18)30365-6 29880291

[B12] Coleman N. Moyers J. T. Harbery A. Vivanco I. Yap T. A. (2021). Clinical development of AKT inhibitors and associated predictive biomarkers to guide patient treatment in cancer medicine. Pharmacogenomics personalized Med. 14, 1517–1535. eng. Epub 2021/12/04. 10.2147/pgpm.S305068 PMC8630372 34858045

[B13] Corn P. G. Heath E. I. Zurita A. Ramesh N. Xiao L. Sei E. (2019). Cabazitaxel plus carboplatin for the treatment of men with metastatic castration-resistant prostate cancers: a randomised, open-label, phase 1-2 trial. Lancet Oncol. 20 (10), 1432–1443. eng. Epub 2019/09/14. 10.1016/s1470-2045(19)30408-5 31515154 PMC6858999

[B14] Crabb S. J. Griffiths G. Dunkley D. Downs N. Ellis M. Radford M. (2022). Overall survival update for patients with metastatic castration-resistant prostate cancer treated with capivasertib and docetaxel in the phase 2 ProCAID clinical trial [journal article]. Eur. Urol. 82 (5), 512–515. Cited in: Pubmed; PMID CN-02459105. 10.1016/j.eururo.2022.05.019 35688662

[B15] De Bono J. Mateo J. Fizazi K. Saad F. Shore N. Sandhu S. (2020). Olaparib for metastatic castration-resistant prostate cancer. N. Engl. J. Med. 382 (22), 2091–2102. 10.1056/NEJMoa1911440 32343890

[B16] de Bono J. S. De Giorgi U. Rodrigues D. N. Massard C. Bracarda S. Font A. (2019). Randomized phase II study evaluating akt blockade with ipatasertib, in combination with abiraterone, in patients with metastatic prostate cancer with and without PTEN loss [journal article]. Clin. cancer Res. 25 (3), 928–936. Cited in: Pubmed; PMID CN-01793834. 10.1158/1078-0432.CCR-18-0981 30037818

[B17] De Wit R. De Bono J. Sternberg C. N. Fizazi K. Tombal B. Wülfing C. (2019). Cabazitaxel versus abiraterone or enzalutamide in metastatic prostate cancer. N. Engl. J. Med. 381 (26), 2506–2518. 10.1056/NEJMoa1911206 31566937

[B18] Fitzpatrick J. M. de Wit R. (2014). Taxane mechanisms of action: potential implications for treatment sequencing in metastatic castration-resistant prostate cancer. Eur. Urol. 65 (6), 1198–1204. eng. Epub 2013/08/06. 10.1016/j.eururo.2013.07.022 23910941

[B19] Fizazi K. Drake C. G. Beer T. M. Kwon E. D. Scher H. I. Gerritsen W. R. (2020). Final analysis of the ipilimumab versus placebo following radiotherapy phase III trial in postdocetaxel metastatic castration-resistant prostate cancer identifies an excess of long-term survivors. Eur. Urol. 78 (6), 822–830. Cited in: Pubmed; PMID WOS:000595089400023. 10.1016/j.eururo.2020.07.032 32811715 PMC8428575

[B20] Fizazi K. Jones R. Oudard S. Efstathiou E. Saad F. de Wit R. (2015). Phase III, randomized, double-blind, multicenter trial comparing orteronel (TAK-700) plus prednisone with placebo plus prednisone in patients with metastatic castration-resistant prostate cancer that has progressed during or after docetaxel-based therapy: ELM-PC 5 [Journal article]. J. Clin. Oncol. 33 (7), 723–731. Cited in: Pubmed; PMID CN-01069015. 10.1200/JCO.2014.56.5119 25624429 PMC4879718

[B21] Fizazi K. Piulats J. M. Reaume M. N. Ostler P. McDermott R. Gingerich J. R. (2023). Rucaparib or physician's choice in metastatic prostate cancer. N. Engl. J. Med. 388, 719–732. 10.1056/NEJMoa2214676 36795891 PMC10064172

[B22] Fizazi K. Scher H. I. Molina A. Logothetis C. J. Chi K. N. Jones R. J. (2012). Abiraterone acetate for treatment of metastatic castration-resistant prostate cancer: final overall survival analysis of the COU-AA-301 randomised, double-blind, placebo-controlled phase 3 study. Lancet Oncol. 13 (10), 983–992. Cited in: Pubmed; PMID WOS:000309510600035. 10.1016/s1470-2045(12)70379-0 22995653

[B23] Freedland S. J. Aronson W. J. (2017). Commentary on "integrative clinical genomics of advanced prostate cancer". Robinson D, van Allen EM, Wu YM, Schultz N, Lonigro RJ, Mosquera JM, Montgomery B, Taplin ME, Pritchard CC, Attard G, Beltran H, Abida W, Bradley RK, Vinson J, Cao X, Vats P, Kunju LP, Hussain M, Feng FY, Tomlins SA, Cooney KA, Smith DC, Brennan C, Siddiqui J, Mehra R, Chen Y, Rathkopf DE, Morris MJ, Solomon SB, Durack JC, Reuter VE, Gopalan A, Gao J, Loda M, Lis RT, Bowden M, Balk SP, Gaviola G, Sougnez C, Gupta M, Yu EY, Mostaghel EA, Cheng HH, Mulcahy H, True LD, Plymate SR, Dvinge H, Ferraldeschi R, Flohr P, Miranda S, Zafeiriou Z, Tunariu N, Mateo J, Perez-Lopez R, Demichelis F, Robinson BD, Schiffman M, Nanus DM, Tagawa ST, Sigaras A, Eng KW, Elemento O, Sboner A, Heath EI, Scher HI, Pienta KJ, Kantoff P, de Bono JS, Rubin MA, Nelson PS, Garraway LA, Sawyers CL, Chinnaiyan AM.cell. 21 may 2015;161(5):1215-1228. Urol. Oncol. 35 (8), 535. Cited in: Pubmed; PMID 28623072. 10.1016/j.urolonc.2017.05.010 28623072

[B24] Gasmi A. Roubaud G. Dariane C. Barret E. Beauval J. B. Brureau L. (2021). Overview of the development and use of akt inhibitors in prostate cancer. J. Clin. Med. 11 (1), 160. Epub 2022/01/12. 10.3390/jcm11010160 35011901 PMC8745410

[B25] Grüllich C. (2018). Cabozantinib: multi-kinase inhibitor of MET, AXL, RET, and VEGFR2. Recent Results Cancer Res. 211, 67–75. eng. Epub 2018/08/03. 10.1007/978-3-319-91442-8_5 30069760

[B26] Hedley Carr T. Adelman C. Barnicle A. Kozarewa I. Luke S. Lai Z. (2021). Homologous recombination repair gene mutation characterization by liquid biopsy: a phase II trial of olaparib and abiraterone in metastatic castrate-resistant prostate cancer. Cancers 13 (22), 5830. 10.3390/cancers13225830 34830984 PMC8616430

[B27] Hirst C. J. Cabrera C. Kirby M. (2012). Epidemiology of castration resistant prostate cancer: a longitudinal a nalysis using a UK primary care database. Cancer Epidemiol. 36 (6), e349–e353. 10.1016/j.canep.2012.07.012 22910034

[B28] Hussain M. Mateo J. Fizazi K. Saad F. Shore N. Sandhu S. (2020). Survival with olaparib in metastatic castration-resistant prostate cancer. N. Engl. J. Med. 383 (24), 2345–2357. 10.1056/NEJMoa2022485 32955174

[B29] Kirby M. Hirst C. Crawford E. D. (2011). Characterising the castration-resistant prostate cancer population: a systematic review. Int. J. Clin. Pract. 65 (11), 1180–1192. 10.1111/j.1742-1241.2011.02799.x 21995694

[B30] Kluetz P. G. Ning Y. M. Maher V. E. Zhang L. Tang S. Ghosh D. (2013). Abiraterone acetate in combination with prednisone for the treatment of patients with metastatic castration-resistant prostate cancer: U.S. Food and Drug Administration drug approval summary. Clin. Cancer Res. 19 (24), 6650–6656. zytiga(Janssen Biotech). 10.1158/1078-0432.CCR-13-2134 24150234

[B31] Liu Z. Zhu G. Getzenberg R. H. Veltri R. W. (2015). The upregulation of PI3K/akt and MAP kinase pathways is associated with resistance of microtubule-targeting drugs in prostate cancer. J. Cell. Biochem. 116 (7), 1341–1349. eng. Epub 2015/02/03. 10.1002/jcb.25091 25640606

[B32] Madan R. A. Karzai F. H. Al Harthy M. Petrylak D. P. Kim J. W. Arlen P. M. (2021). Cabozantinib plus docetaxel and prednisone in metastatic castration-resistant prostate cancer. BJU Int. 127 (4), 435–444. 10.1111/bju.15227 32969563 PMC8265825

[B33] Mateo J. Porta N. Bianchini D. McGovern U. Elliott T. Jones R. (2020). Olaparib in patients with metastatic castration-resistant prostate cancer with DNA repair gene aberrations (TOPARP-B): a multicentre, open-label, randomised, phase 2 trial. Lancet Oncol. 21 (1), 162–174. eng. Epub 2019/12/07. 10.1016/s1470-2045(19)30684-9 31806540 PMC6941219

[B34] Merseburger A. S. Waldron N. Ribal M. J. Heidenreich A. Perner S. Fizazi K. (2021). Genomic testing in patients with metastatic castration-resistant prostate cancer: a pragmatic guide for clinicians. Eur. Urol. 79 (4), 519–529. eng. Epub 2021/01/27. 10.1016/j.eururo.2020.12.039 33494937

[B35] Miller K. Carles J. Gschwend J. E. Van Poppel H. Diels J. Brookman-May S. D. (2018). The phase 3 COU-AA-302 study of abiraterone acetate plus prednisone in men with chemotherapy-naïve metastatic castration-resistant prostate cancer: stratified analysis based on pain, prostate-specific antigen, and gleason score. Eur. Urol. 74 (1), 17–23. eng. Epub 2017/09/25. 10.1016/j.eururo.2017.08.035 28939004

[B36] Monk P. Liu G. Stadler W. M. Geyer S. Huang Y. Wright J. (2018). Phase II randomized, double-blind, placebo-controlled study of tivantinib in men with asymptomatic or minimally symptomatic metastatic castration-resistant prostate cancer (mCRPC). Investig. New Drugs 36 (5), 919–926. Cited in: Pubmed; PMID WOS:000444475200019. 10.1007/s10637-018-0630-9 30083962 PMC6153554

[B37] Nuhn P. De Bono J. S. Fizazi K. Freedland S. J. Grilli M. Kantoff P. W. (2019). Update on systemic prostate cancer therapies: management of metastatic castration-resistant prostate cancer in the era of precision oncology. Eur. Urol. 75 (1), 88–99. eng. Epub 2018/04/21. 10.1016/j.eururo.2018.03.028 29673712

[B38] O'Connor M. J. (2015). Targeting the DNA damage response in cancer. Mol. Cell 60 (4), 547–560. eng. Epub 2015/11/23. 10.1016/j.molcel.2015.10.040 26590714

[B39] Palapattu G. S. (2016). Commentary on "AR-V7 and resistance to enzalutamide and abiraterone in prostate cancer." Antonarakis ES, Lu C, Wang H, Luber B, Nakazawa M, Roeser JC, Chen Y, Mohammad TA, Chen Y, Fedor HL, Lotan TL, Zheng Q, de Marzo AM, Isaacs JT, Isaacs WB, Nadal R, Paller CJ, Denmeade SR, Carducci MA, Eisenberger MA, Luo J, division of urologic oncology, department of Urology, university of Michigan, MI. N engl J med 2014; 371(11):1028-38. Urol. Oncol. 34 (11), 520. 10.1016/j.urolonc.2015.12.011 26964769

[B40] Poorthuis M. H. F. Vernooij R. W. M. van Moorselaar R. J. A. de Reijke T. M. (2017). First-line non-cytotoxic therapy in chemotherapy-naive patients with metastatic castration-resistant prostate cancer: a systematic review of 10 randomised clinical trials. BJU Int. 119 (6), 831–845. eng. Epub 2017/01/08. 10.1111/bju.13764 28063195

[B41] Powles T. Yuen K. C. Gillessen S. Kadel E. E. Rathkopf D. Matsubara N. (2022). Atezolizumab with enzalutamide versus enzalutamide alone in metastatic castration-resistant prostate cancer: a randomized phase 3 trial. Nat. Med. 28 (1), 144–153. Cited in: Pubmed; PMID WOS:000741630800003. 10.1038/s41591-021-01600-6 35013615 PMC9406237

[B42] Rathkopf D. E. Smith M. R. De Bono J. S. Logothetis C. J. Shore N. D. De Souza P. (2014). Updated interim efficacy analysis and long-term safety of abiraterone acetate in metastatic castration-resistant prostate cancer patients without prior chemotherapy (COU-AA-302). Eur. Urol. 66 (5), 815–825. 10.1016/j.eururo.2014.02.056 24647231 PMC4418928

[B43] Rescigno P. Lorente D. Dolling D. Ferraldeschi R. Rodrigues D. N. Riisnaes R. (2018). Docetaxel treatment in PTEN- and ERG-aberrant metastatic prostate cancers. Eur. Urol. Oncol. 1 (1), 71–77. eng. Epub 2018/06/19. 10.1016/j.euo.2018.02.006 29911685 PMC5995869

[B44] Rizzo A. Mollica V. Merler S. Morelli F. Sorgentoni G. Oderda M. (2022). Incidence of grade 3-4 adverse events, dose reduction, and treatment discontinuation in castration-resistant prostate cancer patients receiving PARP inhibitors: a meta-analysis. Expert Opin. drug metabolism Toxicol. 18 (3), 235–240. eng. Epub 2022/04/30. 10.1080/17425255.2022.2072727 35485878

[B45] Ryan C. J. Smith M. R. Fizazi K. Saad F. Mulders P. F. A. Sternberg C. N. (2015). Abiraterone acetate plus prednisone versus placebo plus prednisone in chemotherapy-naive men with metastatic castration-resistant prostate cancer (COU-AA-302): final overall survival analysis of a randomised, double-blind, placebo-controlled phase 3 study. Lancet Oncol. 16 (2), 152–160. 10.1016/S1470-2045(14)71205-7 25601341

[B46] Saad F. Efstathiou E. Attard G. Flaig T. W. Franke F. Goodman O. B. (2021). Apalutamide plus abiraterone acetate and prednisone versus placebo plus abiraterone and prednisone in metastatic, castration-resistant prostate cancer (ACIS): a randomised, placebo-controlled, double-blind, multinational, phase 3 study [Journal Article; Clinical Trial Protocol]. lancet Oncol. 22 (11), 1541–1559. Cited in: Pubmed; PMID CN-02337018. 10.1016/S1470-2045(21)00402-2 34600602 PMC9377412

[B47] Saad F. Fizazi K. Jinga V. Efstathiou E. Fong P. C. Hart L. L. (2015). Orteronel plus prednisone in patients with chemotherapy-naive metastatic castration-resistant prostate cancer (ELM-PC 4): a double-blind, multicentre, phase 3, randomised, placebo-controlled trial. Lancet Oncol. 16 (3), 338–348. tak 700(Takeda,Japan). 10.1016/S1470-2045(15)70027-6 25701170

[B48] Shamseer L. Moher D. Clarke M. Ghersi D. Liberati A. Petticrew M. (2015). Preferred reporting items for systematic review and meta-analysis protocols (PRISMA-P) 2015: elaboration and explanation. BMJ Clin. Res. ed) 350, g7647. 350:g7647. eng. Epub 2015/01/04. 10.1136/bmj.g7647 25555855

[B49] Siegel R. L. Miller K. D. Wagle N. S. Jemal A. (2023). Cancer statistics. CA Cancer J. Clin. 73 (1), 17–30. 10.3322/caac.21332 36633525

[B50] Smith M. De Bono J. Sternberg C. Le Moulec S. Oudard S. De Giorgi U. (2016). Phase III study of cabozantinib in previously treated metastatic castration-resistant prostate cancer: COMET-1. J. Clin. Oncol. 34 (25), 3005–3013. eng. Epub 2016/07/13. 10.1200/jco.2015.65.5597 27400947

[B51] Smith M. R. Kabbinavar F. Saad F. Hussain A. Gittelman M. C. Bilhartz D. L. (2005). Natural history of rising serum prostate-specific antigen in men with castrate nonmetastatic prostate cancer. J. Clin. Oncol. 23 (13), 2918–2925. 10.1200/JCO.2005.01.529 15860850

[B52] Sternberg C. N. Castellano D. de Bono J. Fizazi K. Tombal B. Wülfing C. (2021). Efficacy and safety of cabazitaxel versus abiraterone or enzalutamide in older patients with metastatic castration-resistant prostate cancer in the CARD study. Eur. Urol. 80 (4), 497–506. 10.1016/j.eururo.2021.06.021 34274136

[B53] Sweeney C. Bracarda S. Sternberg C. N. Chi K. N. Olmos D. Sandhu S. (2021). Ipatasertib plus abiraterone and prednisolone in metastatic castration-resistant prostate cancer (IPATential150): a multicentre, randomised, double-blind, phase 3 trial [Journal Article; Clinical Trial Protocol]. Lancet (London, Engl. 398 (10295), 131‐142. Cited in: Pubmed; PMID CN-02292203. 10.1016/S0140-6736(21)00580-8 34246347

[B54] Teyssonneau D. Margot H. Cabart M. Anonnay M. Sargos P. Vuong N. S. (2021). Prostate cancer and PARP inhibitors: progress and challenges. J. Hematol. Oncol. 14 (1), 51. Cited in: Pubmed; PMID 33781305. 10.1186/s13045-021-01061-x 33781305 PMC8008655

[B55] Vogelzang N. J. Beer T. M. Gerritsen W. Oudard S. Wiechno P. Kukielka-Budny B. (2022). Efficacy and safety of autologous dendritic cell-based immunotherapy, docetaxel, and prednisone vs placebo in patients with metastatic castration-resistant prostate cancer: the VIABLE phase 3 randomized clinical trial. JAMA Oncol. 8 (4), 546–552. 10.1001/jamaoncol.2021.7298 35142815 PMC8832307

[B56] Walsh C. S. (2015). Two decades beyond BRCA1/2: homologous recombination, hereditary cancer risk and a target for ovarian cancer therapy. Gynecol. Oncol. 137 (2), 343–350. eng. Epub 2015/03/01. 10.1016/j.ygyno.2015.02.017 25725131

[B57] Wang Y. Zhang H. Shen W. He P. Zhou Z. (2018). Effectiveness and tolerability of targeted drugs for the treatment of metastatic castration-resistant prostate cancer: a network meta-analysis of randomized controlled trials. J. Cancer Res. Clin. Oncol. 144 (9), 1751–1768. eng. Epub 2018/05/26. 10.1007/s00432-018-2664-y 29797220 PMC11813290

[B58] Zarour L. Alumkal J. (2010). Emerging therapies in castrate-resistant prostate cancer. Curr. Urol. Rep. 11 (3), 152–158. 10.1007/s11934-010-0104-x 20425621 PMC3682789

[B59] Zhang Y. Sun M. Huang G. Yin L. Lai Q. Yang Y. (2017). Maintenance of antiangiogenic and antitumor effects by orally active low-dose capecitabine for long-term cancer therapy. Proc. Natl. Acad. Sci. U. S. A. 114 (26), E5226-E5235–e5235. eng. Epub 2017/06/14. 10.1073/pnas.1705066114 28607065 PMC5495268

