# Supplementary material for: Comparing efficacy of first-line treatment of metastatic castration resistant prostate cancer: a network meta-analysis of randomized controlled trials
Source: Front Pharmacol. 2023 Nov 22;14:1290990. doi: 10.3389/fphar.2023.1290990 (PMC10702556; doi:10.3389/fphar.2023.1290990)
Supplement: Supplementary file 1 [file DataSheet1.docx]

Supplementary Table 1A. Matrix of pairwise comparisons of regimens on 3 month overall survival (shown as odds ratio and 95% confidence intervals).

| 1 | castratepreparp | . | . | . | . | . | . | . | . | . | 4.18 (0.46; 38.35) | . | . | . | . | . |
| --- | --- | --- | --- | --- | --- | --- | --- | --- | --- | --- | --- | --- | --- | --- | --- | --- |
| 2 | 1.70 (0.14; 20.69) | chempre | 0.50 (0.01; 25.36) | . | 1.00 (0.18; 5.60) | 0.96 (0.06; 15.87) | . | . | 1.60 (1.07; 2.38) | . | 2.46 (0.78; 7.81) | . | . | . | . | . |
| 3 | 0.85 (0.01; 89.30) | 0.50 (0.01; 25.36) | chempreDCVAC | . | . | . | . | . | . | . | . | . | . | . | . | . |
| 4 | 2.17 (0.22; 21.76) | 1.28 (0.34; 4.77) | 2.54 (0.04; 159.19) | castratepre400ipa | . | . | . | . | . | . | 1.93 (1.02; 3.63) | . | . | . | . | . |
| 5 | 1.70 (0.08; 35.39) | 1.00 (0.18; 5.60) | 1.99 (0.03; 144.48) | 0.78 (0.09; 6.85) | chemprept | . | . | . | . | . | . | . | . | . | . | . |
| 6 | 1.64 (0.04; 69.93) | 0.96 (0.06; 15.87) | 1.92 (0.02; 237.72) | 0.75 (0.03; 16.69) | 0.96 (0.04; 25.76) | chempretarget | . | . | . | . | . | . | . | . | . | . |
| 7 | 2.73 (0.25; 29.97) | 1.61 (0.37; 6.98) | 3.20 (0.05; 210.82) | 1.26 (0.42; 3.81) | 1.60 (0.17; 15.41) | 1.67 (0.07; 39.51) | PD1 | . | . | . | 1.53 (0.62; 3.79) | . | . | . | . | . |
| 8 | 2.57 (0.09; 70.02) | 1.51 (0.10; 22.73) | 3.01 (0.03; 353.80) | 1.18 (0.09; 14.91) | 1.51 (0.06; 37.40) | 1.57 (0.03; 77.45) | 0.94 (0.07; 12.85) | PD1pre | . | . | . | . | . | . | . | 2.14 (0.19; 24.25) |
| 9 | 2.72 (0.22; 34.13) | 1.60 (1.07; 2.38) | 3.18 (0.06; 163.94) | 1.25 (0.32; 4.96) | 1.60 (0.27; 9.33) | 1.66 (0.10; 28.15) | 0.99 (0.22; 4.56) | 1.06 (0.07; 16.40) | chemprePD1 | . | . | . | . | . | . | . |
| 10 | 3.64 (0.37; 36.07) | 2.14 (0.58; 7.83) | 4.26 (0.07; 265.19) | 1.68 (0.70; 3.99) | 2.14 (0.25; 18.43) | 2.22 (0.10; 48.77) | 1.33 (0.45; 3.94) | 1.42 (0.11; 17.68) | 1.34 (0.34; 5.20) | castrateprepd1 | 1.15 (0.64; 2.08) | . | . | . | . | . |
| 11 | 4.18 (0.46; 38.35) | 2.46 (0.78; 7.81) | 4.90 (0.08; 292.06) | 1.93 (1.02; 3.63) | 2.45 (0.31; 19.50) | 2.55 (0.12; 52.93) | 1.53 (0.62; 3.79) | 1.63 (0.14; 18.94) | 1.54 (0.45; 5.22) | 1.15 (0.64; 2.08) | castratepre | . | 1.19 (0.65; 2.18) | 1.50 (0.25; 9.00) | . | 1.31 (0.92; 1.87) |
| 12 | 4.40 (0.45; 43.04) | 2.59 (0.73; 9.24) | 5.16 (0.08; 318.52) | 2.03 (0.89; 4.65) | 2.59 (0.30; 21.97) | 2.69 (0.12; 58.42) | 1.61 (0.56; 4.62) | 1.72 (0.15; 20.08) | 1.62 (0.43; 6.15) | 1.21 (0.55; 2.68) | 1.05 (0.62; 1.79) | target | . | . | . | 1.25 (0.84; 1.85) |
| 13 | 4.97 (0.50; 49.43) | 2.92 (0.79; 10.77) | 5.82 (0.09; 362.93) | 2.29 (0.95; 5.50) | 2.92 (0.34; 25.27) | 3.04 (0.14; 66.79) | 1.82 (0.61; 5.42) | 1.94 (0.15; 24.22) | 1.83 (0.47; 7.15) | 1.37 (0.59; 3.18) | 1.19 (0.65; 2.18) | 1.13 (0.50; 2.52) | castratepre200ipa | . | . | . |
| 14 | 6.26 (0.36; 108.31) | 3.68 (0.44; 31.08) | 7.33 (0.08; 636.83) | 2.88 (0.43; 19.33) | 3.67 (0.24; 56.95) | 3.82 (0.11; 129.45) | 2.29 (0.31; 17.10) | 2.44 (0.12; 50.92) | 2.30 (0.26; 20.18) | 1.72 (0.26; 11.37) | 1.50 (0.25; 9.00) | 1.42 (0.22; 9.23) | 1.26 (0.19; 8.36) | 2castratepre | . | . |
| 15 | 5.35 (0.55; 52.35) | 3.15 (0.88; 11.24) | 6.27 (0.10; 387.35) | 2.47 (1.08; 5.65) | 3.15 (0.37; 26.72) | 3.27 (0.15; 71.05) | 1.96 (0.68; 5.62) | 2.09 (0.18; 24.42) | 1.97 (0.52; 7.48) | 1.47 (0.66; 3.27) | 1.28 (0.75; 2.18) | 1.22 (0.69; 2.13) | 1.08 (0.48; 2.41) | 0.86 (0.13; 5.56) | targetpre | 1.03 (0.69; 1.53) |
| 16 | 5.49 (0.58; 51.85) | 3.23 (0.97; 10.82) | 6.44 (0.11; 389.75) | 2.53 (1.22; 5.24) | 3.23 (0.39; 26.41) | 3.36 (0.16; 71.02) | 2.01 (0.76; 5.33) | 2.14 (0.19; 24.25) | 2.02 (0.57; 7.22) | 1.51 (0.76; 3.01) | 1.31 (0.92; 1.87) | 1.25 (0.84; 1.85) | 1.11 (0.55; 2.23) | 0.88 (0.14; 5.46) | 1.03 (0.69; 1.53) | prednisone |

Supplementary Table 1B. Matrix of pairwise comparisons of regimens on 6 month overall survival (shown as odds ratio and 95% confidence intervals)

| 1 | 2castratepre | . | . | . | . | . | . | . | . | . | 3.06 (0.83; 11.24) | . | . | . | . | . |
| --- | --- | --- | --- | --- | --- | --- | --- | --- | --- | --- | --- | --- | --- | --- | --- | --- |
| 2 | 1.27 (0.20; 7.83) | castratepreparp | . | . | . | . | . | . | . | . | 2.42 (0.67; 8.69) | . | . | . | . | . |
| 3 | 1.57 (0.31; 7.86) | 1.24 (0.25; 6.10) | PD1 | . | . | . | . | . | . | . | 1.95 (0.75; 5.06) | . | . | . | . | . |
| 4 | 2.02 (0.47; 8.63) | 1.59 (0.38; 6.68) | 1.29 (0.41; 4.07) | chempre | 0.92 (0.32; 2.60) | . | 0.95 (0.16; 5.82) | 1.27 (0.57; 2.79) | 1.30 (0.65; 2.61) | . | 1.52 (0.79; 2.90) | . | . | . | . | . |
| 5 | 1.85 (0.31; 11.06) | 1.46 (0.25; 8.60) | 1.18 (0.25; 5.57) | 0.92 (0.32; 2.60) | chemprept | . | . | . | . | . | . | . | . | . | . | . |
| 6 | 2.12 (0.44; 10.22) | 1.67 (0.35; 7.93) | 1.35 (0.37; 4.96) | 1.05 (0.35; 3.15) | 1.14 (0.25; 5.19) | castratepre400ipa | . | . | . | . | 1.45 (0.60; 3.52) | . | . | . | . | . |
| 7 | 1.92 (0.19; 19.57) | 1.52 (0.15; 15.28) | 1.23 (0.14; 10.47) | 0.95 (0.16; 5.82) | 1.04 (0.13; 8.37) | 0.91 (0.11; 7.54) | chempretarget | . | . | . | . | . | . | . | . | . |
| 8 | 2.55 (0.49; 13.35) | 2.02 (0.39; 10.37) | 1.63 (0.40; 6.58) | 1.27 (0.57; 2.79) | 1.38 (0.37; 5.10) | 1.21 (0.31; 4.67) | 1.33 (0.18; 9.57) | chempreDCVAC | . | . | . | . | . | . | . | . |
| 9 | 2.63 (0.53; 13.16) | 2.08 (0.42; 10.21) | 1.68 (0.44; 6.43) | 1.30 (0.65; 2.61) | 1.42 (0.41; 4.96) | 1.24 (0.34; 4.56) | 1.37 (0.20; 9.50) | 1.03 (0.36; 2.95) | chemprePD1 | . | . | . | . | . | . | . |
| 10 | 3.02 (0.62; 14.61) | 2.38 (0.50; 11.34) | 1.92 (0.52; 7.10) | 1.49 (0.50; 4.51) | 1.63 (0.36; 7.42) | 1.42 (0.40; 5.02) | 1.57 (0.19; 13.06) | 1.18 (0.30; 4.59) | 1.15 (0.31; 4.22) | castratepre200ipa | 1.02 (0.42; 2.48) | . | . | . | . | . |
| 11 | 3.06 (0.83; 11.24) | 2.42 (0.67; 8.69) | 1.95 (0.75; 5.06) | 1.52 (0.79; 2.90) | 1.65 (0.49; 5.63) | 1.45 (0.60; 3.52) | 1.59 (0.23; 10.88) | 1.20 (0.43; 3.33) | 1.16 (0.45; 3.01) | 1.02 (0.42; 2.48) | castratepre | 1.02 (0.49; 2.13) | . | . | . | 1.85 (1.23; 2.77) |
| 12 | 3.11 (0.70; 13.88) | 2.46 (0.56; 10.76) | 1.98 (0.59; 6.62) | 1.54 (0.58; 4.12) | 1.68 (0.40; 7.03) | 1.47 (0.46; 4.67) | 1.62 (0.21; 12.68) | 1.22 (0.35; 4.30) | 1.18 (0.36; 3.94) | 1.03 (0.32; 3.29) | 1.02 (0.49; 2.13) | castrateprepd1 | . | . | . | . |
| 13 | 3.76 (0.64; 22.19) | 2.97 (0.51; 17.25) | 2.40 (0.52; 11.17) | 1.86 (0.47; 7.34) | 2.03 (0.36; 11.35) | 1.78 (0.40; 7.96) | 1.96 (0.20; 18.93) | 1.47 (0.30; 7.17) | 1.43 (0.31; 6.65) | 1.25 (0.28; 5.60) | 1.23 (0.37; 4.11) | 1.21 (0.29; 4.98) | PD1pre | . | . | 1.50 (0.48; 4.69) |
| 14 | 4.57 (1.07; 19.57) | 3.61 (0.86; 15.16) | 2.91 (0.92; 9.24) | 2.26 (0.90; 5.68) | 2.47 (0.61; 9.89) | 2.16 (0.72; 6.50) | 2.37 (0.31; 18.07) | 1.79 (0.53; 6.01) | 1.74 (0.55; 5.50) | 1.51 (0.50; 4.58) | 1.49 (0.78; 2.87) | 1.47 (0.55; 3.93) | 1.21 (0.35; 4.23) | targetpre | . | 1.24 (0.74; 2.07) |
| 15 | 4.73 (1.03; 21.74) | 3.73 (0.83; 16.86) | 3.01 (0.87; 10.44) | 2.34 (0.84; 6.55) | 2.55 (0.59; 11.03) | 2.23 (0.68; 7.37) | 2.46 (0.31; 19.70) | 1.85 (0.51; 6.77) | 1.80 (0.52; 6.21) | 1.57 (0.47; 5.19) | 1.54 (0.69; 3.43) | 1.52 (0.51; 4.51) | 1.26 (0.33; 4.75) | 1.03 (0.44; 2.44) | target | 1.20 (0.60; 2.38) |
| 16 | 5.66 (1.45; 22.08) | 4.47 (1.17; 17.08) | 3.61 (1.28; 10.15) | 2.80 (1.31; 6.02) | 3.05 (0.84; 11.11) | 2.67 (1.01; 7.09) | 2.94 (0.41; 20.96) | 2.21 (0.74; 6.65) | 2.15 (0.77; 6.04) | 1.87 (0.70; 5.00) | 1.85 (1.23; 2.77) | 1.82 (0.78; 4.22) | 1.50 (0.48; 4.69) | 1.24 (0.74; 2.07) | 1.20 (0.60; 2.38) | prednisone |

Supplementary Table 1C. Matrix of pairwise comparisons of regimens on 12 month overall survival (shown as odds ratio and 95% confidence intervals)

| 1 | chempretarget | . | . | . | 1.73 (0.72; 4.12) | . | . | . | . | . | . | . | . | . | . | . |
| --- | --- | --- | --- | --- | --- | --- | --- | --- | --- | --- | --- | --- | --- | --- | --- | --- |
| 2 | 1.34 (0.50; 3.60) | chemprePD1 | . | . | 1.29 (0.81; 2.04) | . | . | . | . | . | . | . | . | . | . | . |
| 3 | 1.34 (0.36; 4.98) | 1.00 (0.33; 2.96) | castratepre400ipa | . | . | . | . | . | . | . | . | 2.15 (0.89; 5.21) | . | . | . | . |
| 4 | 1.41 (0.48; 4.09) | 1.05 (0.48; 2.27) | 1.05 (0.33; 3.37) | chemprept | 1.23 (0.66; 2.28) | . | . | . | . | . | . | . | . | . | . | . |
| 5 | 1.73 (0.72; 4.12) | 1.29 (0.81; 2.04) | 1.29 (0.48; 3.47) | 1.23 (0.66; 2.28) | chempre | . | . | . | 1.16 (0.72; 1.87) | . | . | 1.66 (1.08; 2.58) | . | . | . | . |
| 6 | 1.84 (0.57; 5.94) | 1.37 (0.55; 3.40) | 1.38 (0.46; 4.13) | 1.31 (0.48; 3.56) | 1.07 (0.49; 2.33) | PD1 | . | . | . | . | . | 1.56 (0.82; 2.99) | . | . | . | . |
| 7 | 1.90 (0.61; 5.89) | 1.41 (0.60; 3.34) | 1.42 (0.49; 4.09) | 1.35 (0.52; 3.51) | 1.10 (0.53; 2.27) | 1.03 (0.43; 2.46) | 2castratepre | . | . | . | . | 1.52 (0.85; 2.71) | . | . | . | . |
| 8 | 1.98 (0.52; 7.51) | 1.47 (0.48; 4.48) | 1.48 (0.42; 5.27) | 1.41 (0.43; 4.61) | 1.14 (0.42; 3.15) | 1.07 (0.35; 3.29) | 1.04 (0.35; 3.08) | castratepre200ipa | . | . | . | 1.45 (0.58; 3.62) | . | . | . | . |
| 9 | 2.01 (0.75; 5.42) | 1.50 (0.77; 2.91) | 1.50 (0.50; 4.49) | 1.43 (0.66; 3.12) | 1.16 (0.72; 1.87) | 1.09 (0.44; 2.73) | 1.06 (0.44; 2.53) | 1.02 (0.33; 3.11) | chempreDCVAC | . | . | . | . | . | . | . |
| 10 | 2.88 (0.80; 10.41) | 2.14 (0.75; 6.15) | 2.15 (0.63; 7.29) | 2.05 (0.66; 6.35) | 1.66 (0.65; 4.29) | 1.56 (0.54; 4.52) | 1.52 (0.55; 4.21) | 1.45 (0.42; 5.03) | 1.43 (0.50; 4.13) | castratepreparp | . | 1.00 (0.43; 2.32) | . | . | . | . |
| 11 | 2.95 (0.81; 10.72) | 2.19 (0.76; 6.33) | 2.20 (0.65; 7.50) | 2.10 (0.67; 6.54) | 1.71 (0.66; 4.43) | 1.60 (0.55; 4.66) | 1.55 (0.56; 4.34) | 1.49 (0.43; 5.18) | 1.47 (0.51; 4.25) | 1.02 (0.31; 3.38) | PD1pre | . | . | . | . | 1.54 (0.68; 3.50) |
| 12 | 2.88 (1.09; 7.62) | 2.14 (1.13; 4.05) | 2.15 (0.89; 5.21) | 2.05 (0.96; 4.37) | 1.66 (1.08; 2.58) | 1.56 (0.82; 2.99) | 1.52 (0.85; 2.71) | 1.45 (0.58; 3.62) | 1.43 (0.75; 2.73) | 1.00 (0.43; 2.32) | 0.98 (0.42; 2.28) | castratepre | 1.18 (0.74; 1.88) | . | . | 1.58 (1.28; 1.94) |
| 13 | 3.39 (1.15; 9.98) | 2.52 (1.14; 5.56) | 2.53 (0.93; 6.90) | 2.41 (0.99; 5.88) | 1.96 (1.03; 3.72) | 1.84 (0.82; 4.10) | 1.79 (0.85; 3.77) | 1.71 (0.61; 4.78) | 1.68 (0.76; 3.74) | 1.18 (0.45; 3.08) | 1.15 (0.44; 3.03) | 1.18 (0.74; 1.88) | castrateprepd1 | . | . | . |
| 14 | 3.83 (1.35; 10.87) | 2.85 (1.36; 5.97) | 2.86 (1.09; 7.49) | 2.72 (1.17; 6.35) | 2.21 (1.24; 3.95) | 2.08 (0.98; 4.41) | 2.02 (1.01; 4.04) | 1.93 (0.72; 5.20) | 1.90 (0.90; 4.02) | 1.33 (0.53; 3.34) | 1.30 (0.54; 3.13) | 1.33 (0.91; 1.94) | 1.13 (0.62; 2.07) | targetpre | . | 1.18 (0.86; 1.63) |
| 15 | 4.14 (1.39; 12.34) | 3.08 (1.37; 6.90) | 3.10 (1.12; 8.53) | 2.95 (1.19; 7.28) | 2.40 (1.24; 4.64) | 2.25 (0.99; 5.09) | 2.18 (1.02; 4.68) | 2.09 (0.74; 5.91) | 2.06 (0.91; 4.65) | 1.44 (0.54; 3.82) | 1.40 (0.55; 3.59) | 1.44 (0.88; 2.36) | 1.22 (0.62; 2.42) | 1.08 (0.62; 1.88) | target | 1.10 (0.70; 1.72) |
| 16 | 4.54 (1.68; 12.26) | 3.37 (1.73; 6.59) | 3.39 (1.37; 8.41) | 3.23 (1.47; 7.07) | 2.62 (1.62; 4.25) | 2.46 (1.24; 4.87) | 2.39 (1.29; 4.43) | 2.29 (0.90; 5.84) | 2.25 (1.14; 4.44) | 1.58 (0.66; 3.75) | 1.54 (0.68; 3.50) | 1.58 (1.28; 1.94) | 1.34 (0.80; 2.24) | 1.18 (0.86; 1.63) | 1.10 (0.70; 1.72) | prednisone |

Supplementary Table 1D. Matrix of pairwise comparisons of regimens on 18 month overall survival (shown as odds ratio and 95% confidence intervals)

| 1 | chempretarget | . | . | 2.06 (1.05; 4.06) | . | . | . | . | . | . | . | . | . | . |
| --- | --- | --- | --- | --- | --- | --- | --- | --- | --- | --- | --- | --- | --- | --- |
| 2 | 1.42 (0.65; 3.12) | chemprePD1 | . | 1.45 (0.97; 2.17) | . | . | . | . | . | . | . | . | . | . |
| 3 | 1.75 (0.73; 4.19) | 1.23 (0.62; 2.44) | chemprept | 1.18 (0.68; 2.04) | . | . | . | . | . | . | . | . | . | . |
| 4 | 2.06 (1.05; 4.06) | 1.45 (0.97; 2.17) | 1.18 (0.68; 2.04) | chempre | 1.29 (0.90; 1.85) | . | . | 2.63 (1.71; 4.04) | . | . | . | . | . | . |
| 5 | 2.67 (1.24; 5.74) | 1.88 (1.10; 3.22) | 1.52 (0.79; 2.94) | 1.29 (0.90; 1.85) | chempreDCVAC | . | . | . | . | . | . | . | . | . |
| 6 | 4.08 (1.50; 11.11) | 2.87 (1.24; 6.66) | 2.33 (0.93; 5.85) | 1.98 (0.95; 4.14) | 1.53 (0.67; 3.47) | PD1 | . | 1.33 (0.73; 2.43) | . | . | . | . | . | . |
| 7 | 5.00 (2.04; 12.26) | 3.52 (1.72; 7.17) | 2.85 (1.27; 6.39) | 2.42 (1.34; 4.37) | 1.87 (0.94; 3.73) | 1.22 (0.59; 2.53) | 2castratepre | 1.09 (0.72; 1.63) | . | . | . | . | . | . |
| 8 | 5.43 (2.44; 12.09) | 3.82 (2.13; 6.87) | 3.10 (1.54; 6.22) | 2.63 (1.71; 4.04) | 2.03 (1.16; 3.56) | 1.33 (0.73; 2.43) | 1.09 (0.72; 1.63) | castratepre | 1.06 (0.52; 2.17) | . | . | 1.22 (0.83; 1.79) | 1.41 (1.21; 1.63) | . |
| 9 | 5.76 (1.97; 16.87) | 4.05 (1.60; 10.24) | 3.28 (1.21; 8.94) | 2.79 (1.21; 6.44) | 2.16 (0.87; 5.35) | 1.41 (0.55; 3.60) | 1.15 (0.51; 2.63) | 1.06 (0.52; 2.17) | castratepreparp | . | . | . | . | . |
| 10 | 6.06 (2.59; 14.18) | 4.26 (2.22; 8.19) | 3.45 (1.62; 7.35) | 2.93 (1.75; 4.92) | 2.27 (1.21; 4.25) | 1.48 (0.76; 2.89) | 1.21 (0.74; 1.99) | 1.12 (0.84; 1.49) | 1.05 (0.49; 2.28) | targetpre | . | . | 1.26 (0.99; 1.61) | . |
| 11 | 6.54 (2.64; 16.19) | 4.60 (2.23; 9.50) | 3.73 (1.65; 8.45) | 3.17 (1.73; 5.80) | 2.45 (1.21; 4.94) | 1.60 (0.77; 3.34) | 1.31 (0.73; 2.36) | 1.20 (0.79; 1.84) | 1.14 (0.49; 2.62) | 1.08 (0.68; 1.72) | target | . | 1.17 (0.79; 1.74) | . |
| 12 | 6.63 (2.73; 16.11) | 4.66 (2.31; 9.40) | 3.78 (1.70; 8.38) | 3.21 (1.81; 5.71) | 2.48 (1.26; 4.89) | 1.62 (0.80; 3.31) | 1.33 (0.76; 2.32) | 1.22 (0.83; 1.79) | 1.15 (0.51; 2.60) | 1.09 (0.68; 1.77) | 1.01 (0.57; 1.80) | castrateprepd1 | . | . |
| 13 | 7.64 (3.38; 17.25) | 5.38 (2.94; 9.85) | 4.36 (2.13; 8.90) | 3.70 (2.35; 5.83) | 2.86 (1.60; 5.10) | 1.87 (1.01; 3.48) | 1.53 (0.99; 2.36) | 1.41 (1.21; 1.63) | 1.33 (0.64; 2.76) | 1.26 (0.99; 1.61) | 1.17 (0.79; 1.74) | 1.15 (0.76; 1.74) | prednisone | 1.25 (0.56; 2.82) |
| 14 | 9.58 (3.04; 30.21) | 6.74 (2.45; 18.53) | 5.46 (1.86; 16.08) | 4.64 (1.83; 11.74) | 3.59 (1.33; 9.70) | 2.35 (0.85; 6.50) | 1.92 (0.77; 4.80) | 1.76 (0.77; 4.02) | 1.66 (0.56; 4.96) | 1.58 (0.68; 3.69) | 1.46 (0.59; 3.61) | 1.45 (0.58; 3.59) | 1.25 (0.56; 2.82) | PD1pre |

Supplementary Table 1E. Matrix of pairwise comparisons of regimens on 24 month overall survival (shown as odds ratio and 95% confidence intervals)

| 1 | castratepreparp | 1.12 (0.58; 2.18) | . | . | . | . | . | . | . | . | . | . | . |
| --- | --- | --- | --- | --- | --- | --- | --- | --- | --- | --- | --- | --- | --- |
| 2 | 1.12 (0.58; 2.18) | chempretarget | . | 1.80 (0.97; 3.32) | . | . | . | . | . | . | . | . | . |
| 3 | 1.25 (0.47; 3.29) | 1.11 (0.55; 2.26) | chemprePD1 | 1.62 (1.13; 2.32) | . | . | . | . | . | . | . | . | . |
| 4 | 2.01 (0.82; 4.97) | 1.80 (0.97; 3.32) | 1.62 (1.13; 2.32) | chempre | 1.05 (0.61; 1.81) | 1.11 (0.86; 1.43) | . | 3.04 (1.77; 5.20) | . | . | . | . | . |
| 5 | 2.12 (0.74; 6.07) | 1.89 (0.84; 4.29) | 1.70 (0.89; 3.26) | 1.05 (0.61; 1.81) | chemprept | . | . | . | . | . | . | . | . |
| 6 | 2.24 (0.87; 5.71) | 2.00 (1.03; 3.87) | 1.80 (1.16; 2.79) | 1.11 (0.86; 1.43) | 1.06 (0.58; 1.92) | chempreDCVAC | . | . | . | . | . | . | . |
| 7 | 4.82 (1.43; 16.20) | 4.30 (1.56; 11.87) | 3.87 (1.60; 9.38) | 2.39 (1.07; 5.37) | 2.27 (0.86; 6.01) | 2.16 (0.92; 5.03) | PD1 | 1.27 (0.70; 2.32) | . | . | . | . | . |
| 8 | 6.12 (2.14; 17.52) | 5.46 (2.41; 12.35) | 4.91 (2.57; 9.40) | 3.04 (1.77; 5.20) | 2.89 (1.35; 6.19) | 2.74 (1.51; 4.96) | 1.27 (0.70; 2.32) | castratepre | 1.11 (0.84; 1.47) | . | . | 1.38 (1.25; 1.53) | 1.64 (1.16; 2.32) |
| 9 | 6.80 (2.29; 20.20) | 6.07 (2.56; 14.39) | 5.46 (2.70; 11.06) | 3.38 (1.84; 6.19) | 3.21 (1.42; 7.23) | 3.04 (1.58; 5.87) | 1.41 (0.73; 2.74) | 1.11 (0.84; 1.47) | 2castratepre | . | . | . | . |
| 10 | 7.38 (2.53; 21.52) | 6.59 (2.84; 15.26) | 5.93 (3.01; 11.67) | 3.66 (2.06; 6.50) | 3.48 (1.58; 7.66) | 3.30 (1.76; 6.18) | 1.53 (0.81; 2.88) | 1.21 (0.99; 1.47) | 1.08 (0.77; 1.53) | targetpre | . | 1.14 (0.97; 1.36) | . |
| 11 | 9.00 (2.29; 35.39) | 8.03 (2.43; 26.62) | 7.23 (2.43; 21.50) | 4.47 (1.60; 12.50) | 4.25 (1.33; 13.58) | 4.02 (1.40; 11.61) | 1.87 (0.64; 5.41) | 1.47 (0.61; 3.53) | 1.32 (0.53; 3.32) | 1.22 (0.50; 2.96) | PD1pre | 0.94 (0.39; 2.24) | . |
| 12 | 8.45 (2.94; 24.31) | 7.54 (3.31; 17.17) | 6.78 (3.52; 13.08) | 4.19 (2.42; 7.25) | 3.99 (1.85; 8.61) | 3.78 (2.07; 6.91) | 1.75 (0.95; 3.23) | 1.38 (1.25; 1.53) | 1.24 (0.92; 1.67) | 1.14 (0.97; 1.36) | 0.94 (0.39; 2.24) | prednisone | . |
| 13 | 10.03 (3.31; 30.38) | 8.95 (3.68; 21.75) | 8.05 (3.86; 16.82) | 4.98 (2.62; 9.46) | 4.73 (2.04; 10.95) | 4.48 (2.25; 8.94) | 2.08 (1.04; 4.17) | 1.64 (1.16; 2.32) | 1.47 (0.94; 2.30) | 1.36 (0.91; 2.03) | 1.11 (0.43; 2.86) | 1.19 (0.83; 1.71) | castrateprepd1 |

Supplementary Table 1F. Matrix of pairwise comparisons of regimens on 24 month overall survival (shown as odds ratio and 95% confidence intervals)

| 1 | chempretarget | . | . | . | 2.11 (1.08; 4.13) | . | . | . | . | . | . | . |
| --- | --- | --- | --- | --- | --- | --- | --- | --- | --- | --- | --- | --- |
| 2 | 1.31 (0.60; 2.87) | chemprePD1 | . | . | 1.61 (1.08; 2.41) | . | . | . | . | . | . | . |
| 3 | 1.36 (0.33; 5.66) | 1.04 (0.28; 3.88) | chemprePt | . | 1.54 (0.44; 5.41) | . | . | . | . | . | . | . |
| 4 | 1.98 (0.97; 4.05) | 1.51 (0.95; 2.42) | 1.45 (0.41; 5.20) | chempreDCVAC | 1.06 (0.84; 1.35) | . | . | . | . | . | . | . |
| 5 | 2.11 (1.08; 4.13) | 1.61 (1.08; 2.41) | 1.54 (0.44; 5.41) | 1.06 (0.84; 1.35) | chempre | . | . | 2.44 (1.05; 5.66) | . | . | . | . |
| 6 | 4.54 (1.27; 16.33) | 3.47 (1.09; 11.06) | 3.33 (0.63; 17.50) | 2.29 (0.75; 6.98) | 2.16 (0.73; 6.40) | castratepreparp | . | 1.13 (0.57; 2.25) | . | . | . | . |
| 7 | 4.98 (1.65; 15.06) | 3.80 (1.45; 9.99) | 3.65 (0.79; 16.85) | 2.51 (1.01; 6.24) | 2.36 (0.98; 5.69) | 1.10 (0.53; 2.28) | 2castratepre | 1.03 (0.80; 1.33) | . | . | . | . |
| 8 | 5.14 (1.75; 15.10) | 3.93 (1.55; 9.97) | 3.77 (0.83; 17.04) | 2.59 (1.08; 6.22) | 2.44 (1.05; 5.66) | 1.13 (0.57; 2.25) | 1.03 (0.80; 1.33) | castratepre | . | 1.22 (0.64; 2.31) | 1.29 (1.18; 1.43) | . |
| 9 | 5.89 (1.96; 17.68) | 4.49 (1.72; 11.71) | 4.31 (0.94; 19.82) | 2.97 (1.20; 7.32) | 2.79 (1.17; 6.66) | 1.30 (0.63; 2.67) | 1.18 (0.84; 1.65) | 1.14 (0.92; 1.43) | targetpre | . | 1.13 (0.93; 1.38) | . |
| 10 | 6.25 (1.78; 21.89) | 4.77 (1.54; 14.79) | 4.58 (0.89; 23.60) | 3.15 (1.07; 9.32) | 2.96 (1.03; 8.54) | 1.38 (0.54; 3.53) | 1.25 (0.63; 2.50) | 1.22 (0.64; 2.31) | 1.06 (0.54; 2.09) | PD1 | . | . |
| 11 | 6.66 (2.26; 19.63) | 5.08 (1.99; 12.97) | 4.88 (1.08; 22.13) | 3.36 (1.39; 8.09) | 3.16 (1.35; 7.36) | 1.46 (0.73; 2.94) | 1.34 (1.02; 1.75) | 1.29 (1.18; 1.43) | 1.13 (0.93; 1.38) | 1.07 (0.56; 2.04) | prednisone | 4.10 (1.25; 13.41) |
| 12 | 27.28 (5.48; 135.77) | 20.82 (4.59; 94.39) | 19.99 (2.93; 136.57) | 13.76 (3.14; 60.23) | 12.94 (3.02; 55.55) | 6.00 (1.52; 23.74) | 5.48 (1.62; 18.48) | 5.31 (1.61; 17.43) | 4.63 (1.39; 15.42) | 4.37 (1.13; 16.86) | 4.10 (1.25; 13.41) | PD1pre |

Supplementary Table 2A. Matrix of pairwise comparisons of regimens on 3 month Progression free survival (shown as odds ratio and 95% confidence intervals)

| 1 | chemprept | 2.21 (1.19; 4.12) | . | . | . | . | . | . |
| --- | --- | --- | --- | --- | --- | --- | --- | --- |
| 2 | 2.21 ( 1.19; 4.12) | chempre | . | . | 3.92 (2.58; 5.98) | . | . | . |
| 3 | 4.05 ( 1.58; 10.40) | 1.83 ( 0.90; 3.72) | castratepretarget | . | 2.14 (1.21; 3.79) | . | . | . |
| 4 | 4.42 ( 1.71; 11.45) | 2.00 ( 0.97; 4.11) | 1.09 ( 0.48; 2.47) | parp | 1.96 (1.09; 3.52) | . | . | . |
| 5 | 8.68 ( 4.09; 18.40) | 3.92 ( 2.58; 5.98) | 2.14 ( 1.21; 3.79) | 1.96 ( 1.09; 3.52) | castratepre | . | 2.78 (2.12; 3.63) | . |
| 6 | 15.89 ( 6.72; 37.59) | 7.19 ( 3.96; 13.03) | 3.92 ( 1.93; 7.96) | 3.60 ( 1.75; 7.39) | 1.83 ( 1.20; 2.79) | targetpre | 1.52 (1.10; 2.09) | . |
| 7 | 24.11 (10.85; 53.54) | 10.90 ( 6.61; 17.96) | 5.95 ( 3.17; 11.17) | 5.46 ( 2.87; 10.38) | 2.78 ( 2.12; 3.63) | 1.52 ( 1.10; 2.09) | prednisone | 1.54 (0.74; 3.21) |
| 8 | 37.08 (12.53; 109.75) | 16.77 ( 6.89; 40.79) | 9.15 ( 3.48; 24.11) | 8.39 ( 3.16; 22.29) | 4.27 ( 1.95; 9.35) | 2.33 ( 1.04; 5.21) | 1.54 ( 0.74; 3.21) | PD1pre |

Supplementary Table 2B. Matrix of pairwise comparisons of regimens on 6 month Progression free survival (shown as odds ratio and 95% confidence intervals)

| 1 | chemprept | 1.93 (1.05; 3.55) | . | . | . | . | . | . |
| --- | --- | --- | --- | --- | --- | --- | --- | --- |
| 2 | 1.93 (1.05; 3.55) | chempre | . | . | 2.97 (1.73; 5.09) | . | . | . |
| 3 | 2.01 (0.67; 6.04) | 1.04 (0.42; 2.60) | castratepretarget | . | 2.86 (1.36; 6.00) | . | . | . |
| 4 | 2.45 (0.92; 6.53) | 1.27 (0.59; 2.73) | 1.22 (0.49; 3.07) | parp | 2.34 (1.36; 4.02) | . | . | . |
| 5 | 5.73 (2.54; 12.92) | 2.97 (1.73; 5.09) | 2.86 (1.36; 6.00) | 2.34 (1.36; 4.02) | castratepre | . | 2.63 (1.91; 3.63) | . |
| 6 | 9.92 (3.77; 26.12) | 5.14 (2.42; 10.91) | 4.95 (1.99; 12.27) | 4.04 (1.90; 8.61) | 1.73 (1.02; 2.93) | targetpre | 1.52 (1.00; 2.30) | . |
| 7 | 15.09 (6.30; 36.18) | 7.82 (4.18; 14.65) | 7.52 (3.35; 16.89) | 6.15 (3.27; 11.57) | 2.63 (1.91; 3.63) | 1.52 (1.00; 2.30) | prednisone | 1.53 (0.62; 3.73) |
| 8 | 23.02 (6.59; 80.41) | 11.93 (4.00; 35.57) | 11.47 (3.44; 38.32) | 9.38 (3.14; 28.04) | 4.02 (1.55; 10.39) | 2.32 (0.87; 6.22) | 1.53 (0.62; 3.73) | PD1pre |

Supplementary Table 2C. Matrix of pairwise comparisons of regimens on 12 month Progression free survival (shown as odds ratio and 95% confidence intervals)

| 1 | chempre | 1.12 (0.54; 2.34) | . | . | 4.21 (1.16; 15.28) | . | . | . |
| --- | --- | --- | --- | --- | --- | --- | --- | --- |
| 2 | 1.12 (0.54; 2.34) | chemprept | . | . | . | . | . | . |
| 3 | 1.34 (0.33; 5.39) | 1.19 (0.25; 5.77) | parp | . | 3.14 (1.86; 5.32) | . | . | . |
| 4 | 1.49 (0.31; 7.20) | 1.33 (0.23; 7.56) | 1.12 (0.39; 3.17) | castratepretarget | 2.82 (1.14; 6.95) | . | . | . |
| 5 | 4.21 (1.16; 15.28) | 3.75 (0.85; 16.56) | 3.14 (1.86; 5.32) | 2.82 (1.14; 6.95) | castratepre | . | 2.41 (1.94; 2.98) | . |
| 6 | 5.86 (1.53; 22.40) | 5.23 (1.13; 24.13) | 4.38 (2.31; 8.31) | 3.93 (1.49; 10.39) | 1.39 (0.97; 2.01) | targetpre | 1.73 (1.29; 2.31) | . |
| 7 | 10.12 (2.74; 37.44) | 9.03 (2.01; 40.49) | 7.57 (4.28; 13.37) | 6.78 (2.68; 17.15) | 2.41 (1.94; 2.98) | 1.73 (1.29; 2.31) | prednisone | 3.08 (0.78; 12.25) |
| 8 | 31.23 (4.67; 208.90) | 27.85 (3.63; 213.75) | 23.34 (5.25; 103.74) | 20.93 (3.97; 110.24) | 7.43 (1.84; 29.98) | 5.33 (1.30; 21.81) | 3.08 (0.78; 12.25) | PD1pre |

Supplementary Table 2D. Matrix of pairwise comparisons of regimens on 18 month Progression free survival (shown as odds ratio and 95% confidence intervals)

| 1 | parp | . | . | . | 3.08 (1.67; 5.71) | . | . |
| --- | --- | --- | --- | --- | --- | --- | --- |
| 2 | 1.02 (0.18; 5.95) | chemprept | . | 1.51 (0.64; 3.56) | . | . | . |
| 3 | 1.08 (0.24; 4.88) | 1.06 (0.12; 9.05) | castratepretarget | . | 2.85 (0.72; 11.29) | . | . |
| 4 | 1.54 (0.33; 7.17) | 1.51 (0.64; 3.56) | 1.43 (0.20; 10.21) | chempre | 2.00 (0.49; 8.18) | . | . |
| 5 | 3.08 (1.67; 5.71) | 3.02 (0.58; 15.69) | 2.85 (0.72; 11.29) | 2.00 (0.49; 8.18) | castratepre | . | 2.23 (1.77; 2.81) |
| 6 | 4.75 (2.24; 10.10) | 4.65 (0.84; 25.57) | 4.40 (1.04; 18.60) | 3.08 (0.71; 13.45) | 1.54 (1.00; 2.38) | targetpre | 1.45 (1.00; 2.09) |
| 7 | 6.87 (3.56; 13.27) | 6.72 (1.27; 35.52) | 6.36 (1.58; 25.64) | 4.46 (1.07; 18.57) | 2.23 (1.77; 2.81) | 1.45 (1.00; 2.09) | prednisone |

Supplementary Table 3A. Matrix of pairwise comparisons of regimens on 3 month radiographic Progression free survival (shown as odds ratio and 95% confidence intervals)

| 1 | target | . | . | . | . | . | . | 4.25 (3.23; 5.59) | . | . | . | . |
| --- | --- | --- | --- | --- | --- | --- | --- | --- | --- | --- | --- | --- |
| 2 | 1.66 (0.28; 9.71) | castratepretarget | . | . | . | . | . | . | . | . | . | 4.15 (1.53; 11.25) |
| 3 | 2.30 (0.50; 10.60) | 1.39 (0.47; 4.14) | chempre | . | . | . | . | . | . | . | . | 2.99 (1.91; 4.68) |
| 4 | 2.71 (0.51; 14.32) | 1.64 (0.46; 5.86) | 1.18 (0.47; 2.94) | castratepreparp | . | . | . | . | . | . | . | 2.54 (1.14; 5.63) |
| 5 | 2.72 (1.78; 4.16) | 1.64 (0.28; 9.70) | 1.18 (0.25; 5.51) | 1.00 (0.19; 5.35) | targetpre | . | . | 1.56 (1.13; 2.16) | . | . | . | . |
| 6 | 3.09 (0.67; 14.21) | 1.86 (0.63; 5.55) | 1.34 (0.72; 2.52) | 1.14 (0.46; 2.84) | 1.14 (0.24; 5.28) | PD1 | . | . | . | . | . | 2.23 (1.43; 3.47) |
| 7 | 4.20 (0.90; 19.73) | 2.54 (0.83; 7.76) | 1.83 (0.93; 3.60) | 1.55 (0.60; 3.99) | 1.54 (0.33; 7.32) | 1.36 (0.69; 2.67) | 2castratepre | . | . | . | . | 1.64 (0.98; 2.72) |
| 8 | 4.25 (3.23; 5.59) | 2.57 (0.45; 14.71) | 1.85 (0.41; 8.31) | 1.57 (0.30; 8.09) | 1.56 (1.13; 2.16) | 1.38 (0.31; 6.18) | 1.01 (0.22; 4.63) | prednisone | . | . | . | 1.62 (0.39; 6.79) |
| 9 | 4.75 (1.00; 22.52) | 2.87 (0.92; 8.89) | 2.07 (1.03; 4.16) | 1.75 (0.67; 4.58) | 1.75 (0.36; 8.36) | 1.54 (0.77; 3.09) | 1.13 (0.54; 2.37) | 1.12 (0.24; 5.17) | castratepre400ipa | . | . | 1.45 (0.85; 2.48) |
| 10 | 6.48 (1.46; 28.74) | 3.91 (1.39; 11.05) | 2.82 (1.65; 4.81) | 2.39 (1.02; 5.59) | 2.38 (0.53; 10.67) | 2.10 (1.23; 3.57) | 1.54 (0.86; 2.77) | 1.53 (0.35; 6.59) | 1.36 (0.74; 2.51) | castrateprepd1 | . | 1.06 (0.79; 1.42) |
| 11 | 7.22 (1.43; 36.43) | 4.36 (1.29; 14.71) | 3.14 (1.37; 7.20) | 2.66 (0.92; 7.68) | 2.65 (0.52; 13.51) | 2.34 (1.02; 5.34) | 1.72 (0.72; 4.07) | 1.70 (0.34; 8.37) | 1.52 (0.63; 3.67) | 1.11 (0.52; 2.37) | castratepre200ipa | 0.95 (0.47; 1.92) |
| 12 | 6.88 (1.60; 29.64) | 4.15 (1.53; 11.25) | 2.99 (1.91; 4.68) | 2.54 (1.14; 5.63) | 2.53 (0.58; 11.00) | 2.23 (1.43; 3.47) | 1.64 (0.98; 2.72) | 1.62 (0.39; 6.79) | 1.45 (0.85; 2.48) | 1.06 (0.79; 1.42) | 0.95 (0.47; 1.92) | castratepre |

Supplementary Table 3B. Matrix of pairwise comparisons of regimens on 6 month radiographic Progression free survival (shown as odds ratio and 95% confidence intervals)

| 1 | PD1 | . | . | . | . | . | . | . | 3.08 (1.92; 4.96) | . | . | . |
| --- | --- | --- | --- | --- | --- | --- | --- | --- | --- | --- | --- | --- |
| 2 | 1.20 (0.64; 2.25) | chempre | . | . | . | . | . | . | 2.57 (1.71; 3.86) | . | . | . |
| 3 | 1.37 (0.60; 3.11) | 1.14 (0.52; 2.49) | castratepreparp | . | . | . | . | . | 2.25 (1.16; 4.38) | . | . | . |
| 4 | 1.70 (0.73; 3.96) | 1.41 (0.63; 3.18) | 1.24 (0.47; 3.26) | castratepretarget | . | . | . | . | 1.82 (0.90; 3.67) | . | . | . |
| 5 | 1.78 (0.97; 3.28) | 1.49 (0.85; 2.60) | 1.30 (0.60; 2.81) | 1.05 (0.47; 2.34) | castratepre400ipa | . | . | . | 1.73 (1.18; 2.53) | . | . | . |
| 6 | 1.90 (1.05; 3.42) | 1.58 (0.92; 2.71) | 1.38 (0.65; 2.94) | 1.12 (0.51; 2.45) | 1.06 (0.63; 1.78) | 2castratepre | . | . | 1.62 (1.15; 2.30) | . | . | . |
| 7 | 2.29 (1.05; 4.99) | 1.91 (0.91; 4.00) | 1.67 (0.67; 4.15) | 1.35 (0.53; 3.44) | 1.28 (0.62; 2.65) | 1.21 (0.59; 2.45) | castratepre200ipa | . | 1.35 (0.73; 2.49) | . | . | . |
| 8 | 2.87 (1.64; 5.01) | 2.39 (1.45; 3.94) | 2.09 (1.01; 4.33) | 1.69 (0.79; 3.62) | 1.61 (0.99; 2.60) | 1.51 (0.96; 2.38) | 1.25 (0.63; 2.47) | castrateprepd1 | 1.08 (0.80; 1.44) | . | . | . |
| 9 | 3.08 (1.92; 4.96) | 2.57 (1.71; 3.86) | 2.25 (1.16; 4.38) | 1.82 (0.90; 3.67) | 1.73 (1.18; 2.53) | 1.62 (1.15; 2.30) | 1.35 (0.73; 2.49) | 1.08 (0.80; 1.44) | castratepre | . | . | 7.36 (3.11; 17.43) |
| 10 | 6.72 (2.39; 18.94) | 5.60 (2.05; 15.32) | 4.91 (1.58; 15.27) | 3.97 (1.25; 12.62) | 3.77 (1.39; 10.20) | 3.54 (1.32; 9.48) | 2.93 (0.97; 8.88) | 2.34 (0.89; 6.15) | 2.18 (0.87; 5.47) | target | . | 3.38 (2.45; 4.65) |
| 11 | 11.76 (4.29; 32.27) | 9.80 (3.68; 26.09) | 8.58 (2.82; 26.09) | 6.94 (2.23; 21.56) | 6.59 (2.50; 17.36) | 6.20 (2.38; 16.12) | 5.13 (1.74; 15.15) | 4.10 (1.61; 10.46) | 3.81 (1.57; 9.29) | 1.75 (1.18; 2.58) | targetpre | 1.93 (1.55; 2.41) |
| 12 | 22.69 (8.48; 60.74) | 18.90 (7.28; 49.08) | 16.56 (5.57; 49.23) | 13.39 (4.40; 40.71) | 12.72 (4.95; 32.65) | 11.96 (4.72; 30.31) | 9.90 (3.43; 28.57) | 7.92 (3.19; 19.66) | 7.36 (3.11; 17.43) | 3.38 (2.45; 4.65) | 1.93 (1.55; 2.41) | prednisone |

Supplementary Table 3C. Matrix of pairwise comparisons of regimens on 12 month radiographic Progression free survival (shown as odds ratio and 95% confidence intervals)

| 1 | chempre | . | . | . | . | . | . | . | 3.74 (2.12; 6.59) | . | . | . |
| --- | --- | --- | --- | --- | --- | --- | --- | --- | --- | --- | --- | --- |
| 2 | 1.89 (0.84; 4.26) | castratepreparp | . | . | . | . | . | . | 1.98 (1.10; 3.56) | . | . | . |
| 3 | 2.01 (0.89; 4.55) | 1.07 (0.46; 2.45) | PD1 | . | . | . | . | . | 1.86 (1.03; 3.36) | . | . | . |
| 4 | 2.18 (1.14; 4.18) | 1.16 (0.59; 2.26) | 1.09 (0.56; 2.12) | castratepre400ipa | . | . | . | . | 1.71 (1.24; 2.36) | . | . | . |
| 5 | 2.25 (0.90; 5.62) | 1.19 (0.47; 3.02) | 1.12 (0.44; 2.84) | 1.03 (0.47; 2.27) | castratepretarget | . | . | . | 1.66 (0.81; 3.43) | . | . | . |
| 6 | 2.58 (1.38; 4.82) | 1.37 (0.72; 2.60) | 1.28 (0.67; 2.45) | 1.18 (0.78; 1.79) | 1.15 (0.53; 2.48) | 2castratepre | . | . | 1.45 (1.11; 1.89) | . | . | . |
| 7 | 2.99 (1.23; 7.28) | 1.59 (0.64; 3.91) | 1.49 (0.60; 3.68) | 1.37 (0.64; 2.92) | 1.33 (0.49; 3.60) | 1.16 (0.56; 2.42) | castratepre200ipa | . | 1.25 (0.63; 2.48) | . | . | . |
| 8 | 3.15 (1.56; 6.33) | 1.67 (0.82; 3.41) | 1.57 (0.76; 3.21) | 1.44 (0.86; 2.43) | 1.40 (0.61; 3.21) | 1.22 (0.75; 1.99) | 1.05 (0.47; 2.34) | castrateprepd1 | 1.19 (0.79; 1.79) | . | . | . |
| 9 | 3.74 (2.12; 6.59) | 1.98 (1.10; 3.56) | 1.86 (1.03; 3.36) | 1.71 (1.24; 2.36) | 1.66 (0.81; 3.43) | 1.45 (1.11; 1.89) | 1.25 (0.63; 2.48) | 1.19 (0.79; 1.79) | castratepre | . | . | 2.34 (1.68; 3.26) |
| 10 | 4.35 (1.92; 9.87) | 2.31 (1.00; 5.31) | 2.17 (0.94; 4.99) | 2.00 (1.02; 3.91) | 1.94 (0.76; 4.93) | 1.69 (0.88; 3.23) | 1.46 (0.59; 3.60) | 1.38 (0.67; 2.84) | 1.16 (0.64; 2.10) | target | . | 2.01 (1.23; 3.28) |
| 11 | 4.57 (2.30; 9.08) | 2.42 (1.20; 4.90) | 2.28 (1.12; 4.61) | 2.10 (1.27; 3.46) | 2.04 (0.90; 4.62) | 1.77 (1.11; 2.84) | 1.53 (0.70; 3.36) | 1.45 (0.83; 2.56) | 1.22 (0.83; 1.80) | 1.05 (0.62; 1.78) | targetpre | 1.92 (1.57; 2.34) |
| 12 | 8.76 (4.55; 16.88) | 4.65 (2.37; 9.11) | 4.36 (2.22; 8.58) | 4.02 (2.53; 6.37) | 3.90 (1.76; 8.63) | 3.40 (2.22; 5.20) | 2.93 (1.37; 6.27) | 2.79 (1.64; 4.72) | 2.34 (1.68; 3.26) | 2.01 (1.23; 3.28) | 1.92 (1.57; 2.34) | prednisone |

Supplementary Table 3D. Matrix of pairwise comparisons of regimens on 18 month radiographic Progression free survival (shown as odds ratio and 95% confidence intervals)

| 1 | chempre | . | . | . | . | . | . | . | 2.94 (1.19; 7.26) | . | . | . |
| --- | --- | --- | --- | --- | --- | --- | --- | --- | --- | --- | --- | --- |
| 2 | 1.11 (0.31; 3.94) | castrateprepd1 | . | . | . | . | . | . | 2.64 (1.09; 6.40) | . | . | . |
| 3 | 1.72 (0.50; 5.97) | 1.55 (0.45; 5.29) | castratepretarget | . | . | . | . | . | 1.71 (0.73; 4.00) | . | . | . |
| 4 | 1.86 (0.71; 4.85) | 1.67 (0.65; 4.28) | 1.08 (0.43; 2.68) | castratepre400ipa | . | . | . | . | 1.58 (1.15; 2.17) | . | . | . |
| 5 | 1.93 (0.76; 4.94) | 1.74 (0.69; 4.37) | 1.12 (0.46; 2.73) | 1.04 (0.69; 1.56) | 2castratepre | . | . | . | 1.52 (1.18; 1.95) | . | . | . |
| 6 | 1.90 (0.64; 5.65) | 1.71 (0.58; 5.00) | 1.10 (0.39; 3.14) | 1.02 (0.52; 2.03) | 0.98 (0.51; 1.90) | castratepreparp | . | . | 1.55 (0.84; 2.83) | . | . | . |
| 7 | 2.66 (1.02; 6.95) | 2.39 (0.93; 6.14) | 1.55 (0.62; 3.85) | 1.43 (0.91; 2.26) | 1.38 (0.91; 2.07) | 1.40 (0.70; 2.78) | targetpre | . | . | . | . | 1.86 (1.49; 2.31) |
| 8 | 2.88 (0.85; 9.80) | 2.60 (0.77; 8.70) | 1.68 (0.51; 5.49) | 1.55 (0.64; 3.75) | 1.49 (0.63; 3.53) | 1.52 (0.55; 4.22) | 1.08 (0.45; 2.63) | castratepre200ipa | 1.02 (0.45; 2.32) | . | . | . |
| 9 | 2.94 (1.19; 7.26) | 2.64 (1.09; 6.40) | 1.71 (0.73; 4.00) | 1.58 (1.15; 2.17) | 1.52 (1.18; 1.95) | 1.55 (0.84; 2.83) | 1.10 (0.80; 1.53) | 1.02 (0.45; 2.32) | castratepre | 1.51 (0.73; 3.11) | . | 1.68 (1.32; 2.14) |
| 10 | 4.42 (1.39; 14.09) | 3.98 (1.27; 12.49) | 2.57 (0.84; 7.87) | 2.38 (1.08; 5.25) | 2.29 (1.06; 4.93) | 2.33 (0.90; 5.99) | 1.66 (0.75; 3.68) | 1.53 (0.51; 4.59) | 1.51 (0.73; 3.11) | PD1 | . | . |
| 11 | 4.47 (1.49; 13.39) | 4.02 (1.36; 11.86) | 2.60 (0.90; 7.46) | 2.41 (1.20; 4.84) | 2.31 (1.18; 4.52) | 2.35 (0.99; 5.61) | 1.68 (0.91; 3.11) | 1.55 (0.55; 4.35) | 1.52 (0.82; 2.83) | 1.01 (0.39; 2.63) | target | 1.11 (0.62; 1.96) |
| 12 | 4.94 (1.94; 12.59) | 4.44 (1.78; 11.12) | 2.87 (1.18; 6.96) | 2.66 (1.79; 3.96) | 2.55 (1.81; 3.62) | 2.60 (1.35; 4.99) | 1.86 (1.49; 2.31) | 1.71 (0.73; 4.04) | 1.68 (1.32; 2.14) | 1.12 (0.52; 2.40) | 1.11 (0.62; 1.96) | prednisone |

Supplementary Figure 1A. Convergence of the three Markov Chain Monte Carlo chains established by of the history feature for overall survival.


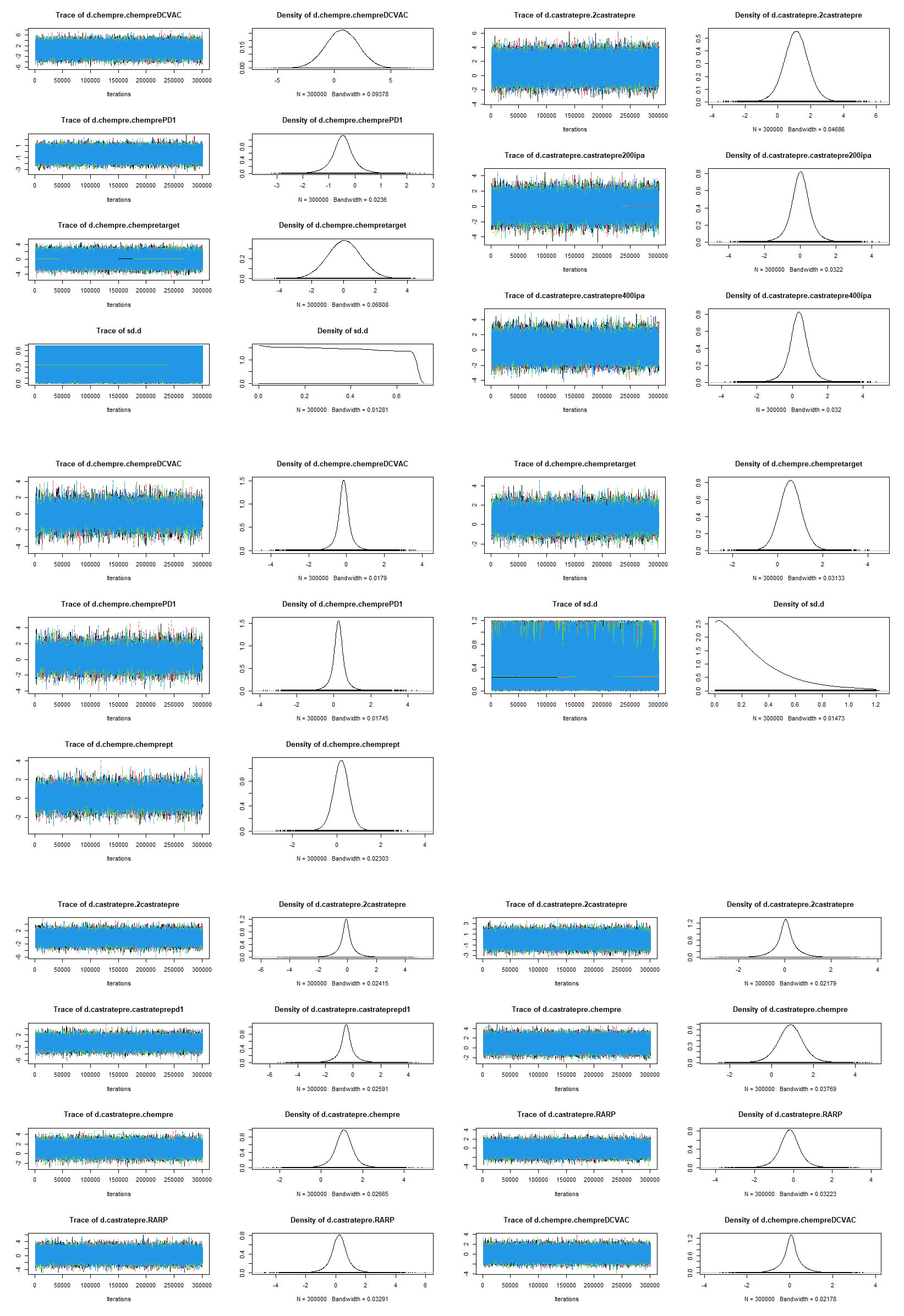


Supplementary Figure 1B. Convergence of the three Markov Chain Monte Carlo chains established by of the history feature for Progression free survival.


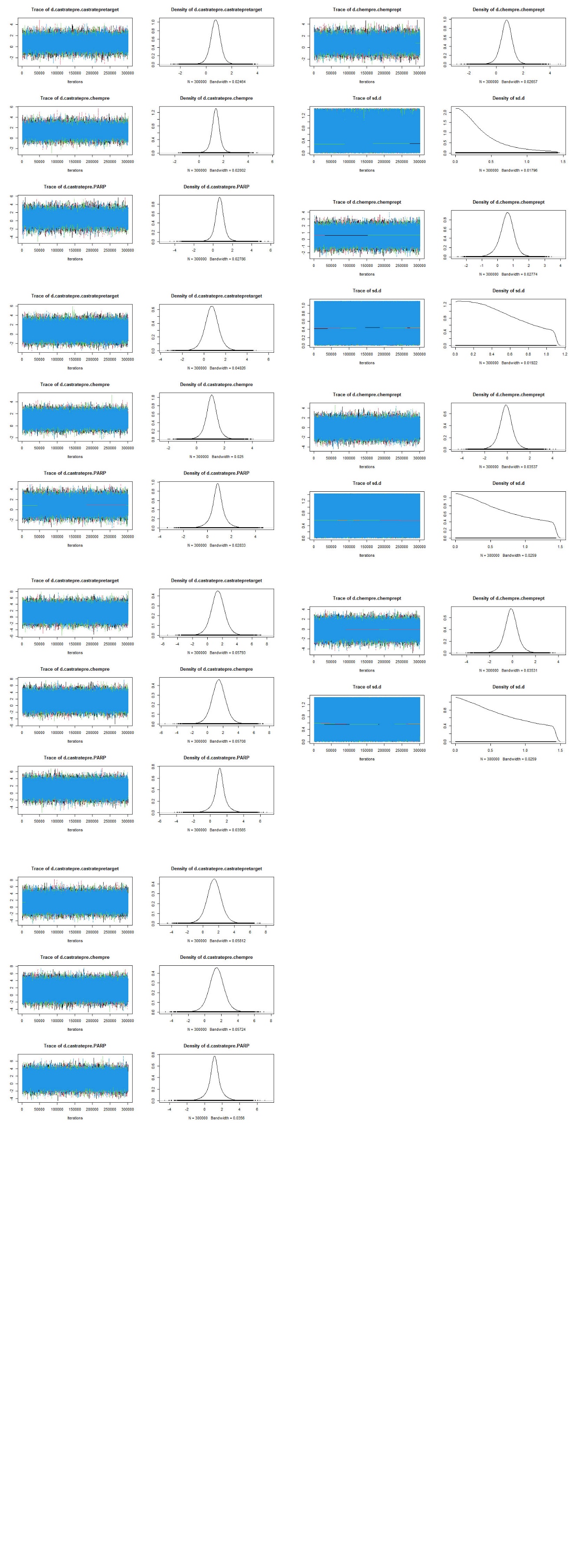


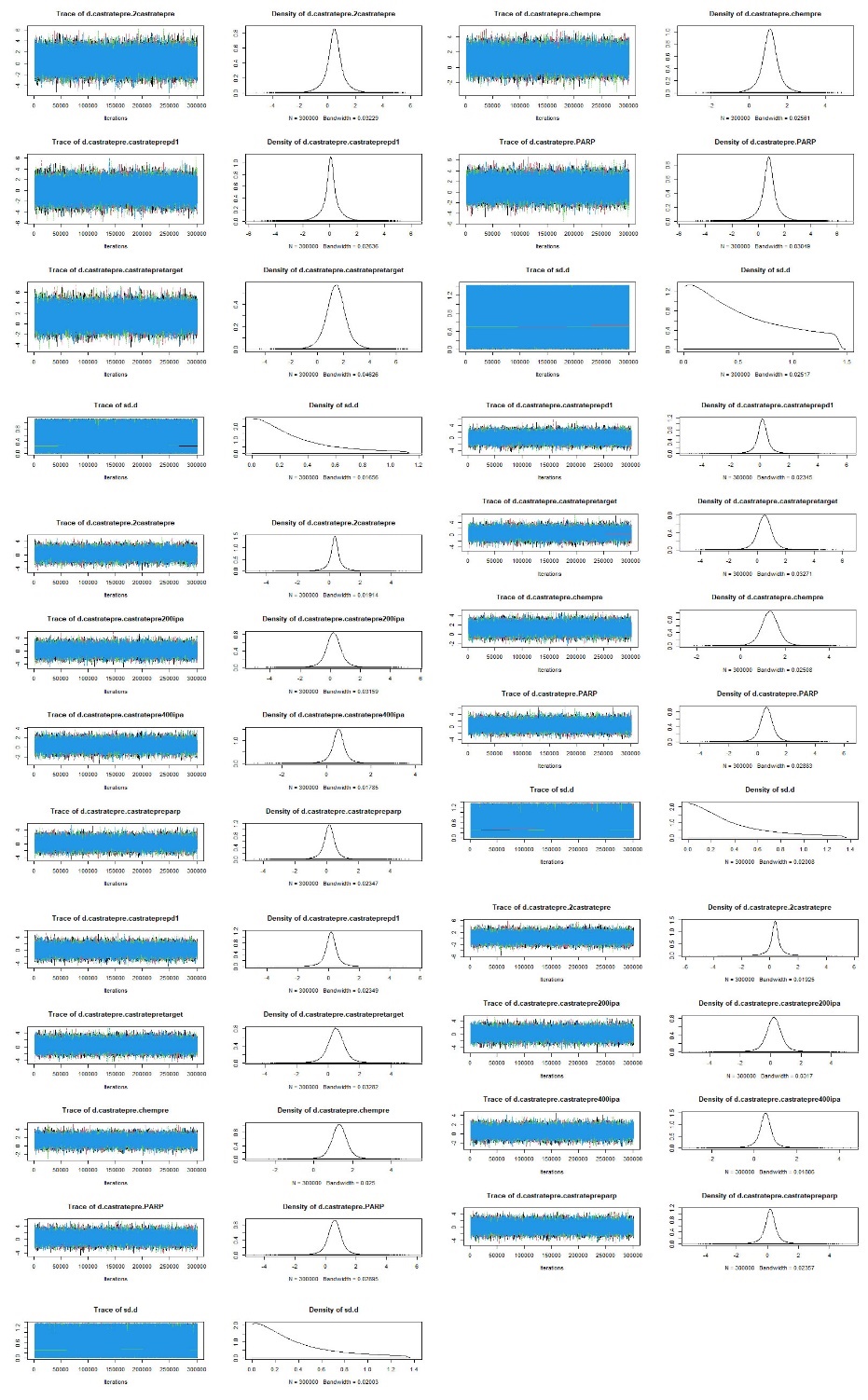
Supplementary Figure 1C. Convergence of the three Markov Chain Monte Carlo chains established by of the history feature for radiographic Progression free survival


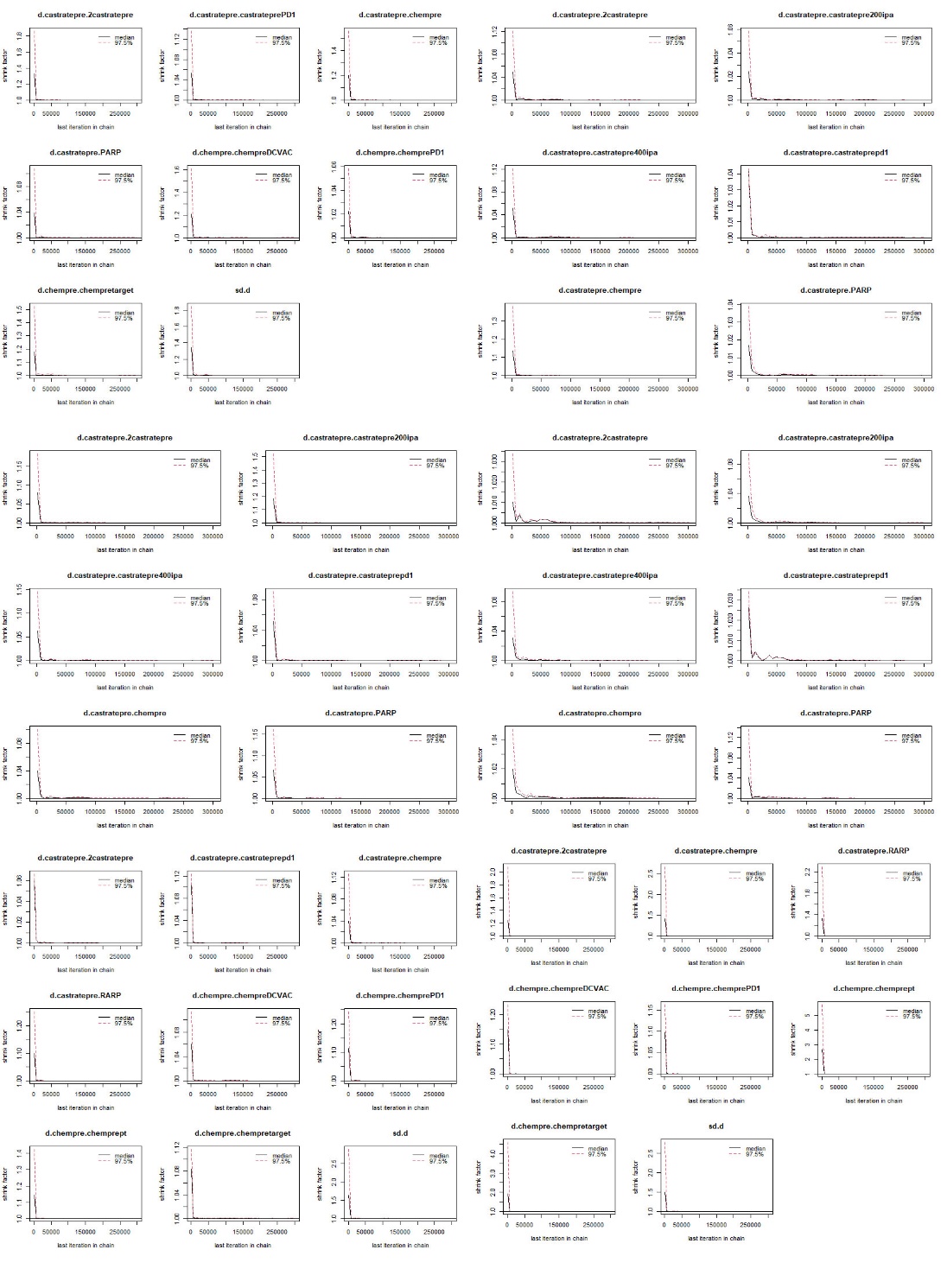
Supplementary Figure 2A. Convergence of the three Markov Chain Monte Carlo chains established by of the Brooks-Gelman-Rubin diagnostic for overall survival


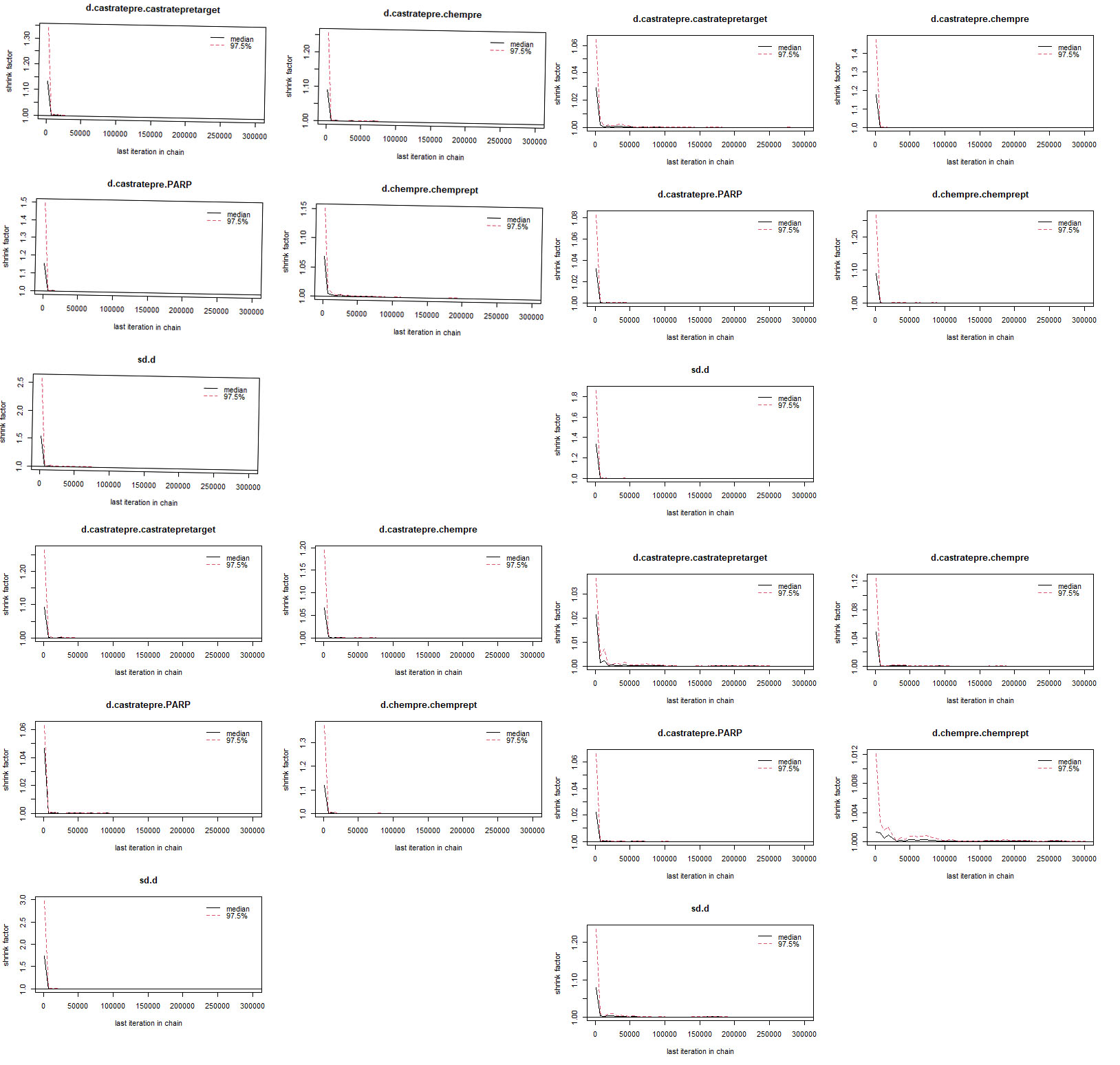


Supplementary Figure 2B. Convergence of the three Markov Chain Monte Carlo chains established by of the Brooks-Gelman-Rubin diagnostic for Progression free survival


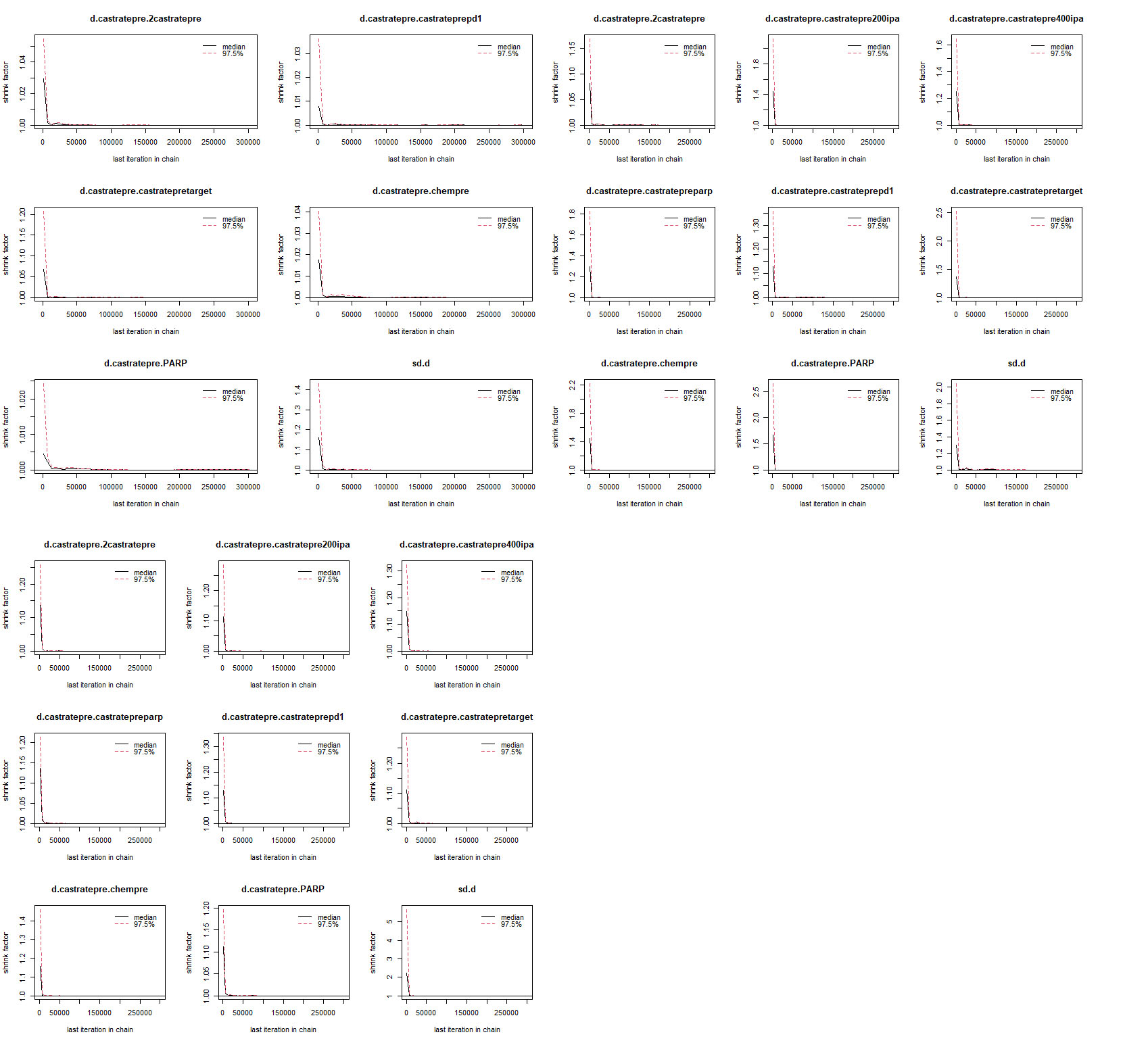


Supplementary Figure 2C. Convergence of the three Markov Chain Monte Carlo chains established by of the Brooks-Gelman-Rubin diagnostic for radiographic Progression free survival
